# Inference and visualization of complex genotype-phenotype maps with *gpmap-tools*

**DOI:** 10.1101/2025.03.09.642267

**Published:** 2025-03-15

**Authors:** Carlos Martí-Gómez, Juannan Zhou, Wei-Chia Chen, Justin B. Kinney, David M. McCandlish

**Affiliations:** 1 Simons Center for Quantitative Biology, Cold Spring Harbor Laboratory, Cold Spring Harbor, NY, 11724; 2 Department of Biology, University of Florida, Gainesville, FL, 32611; 3 Department of Physics, National Chung Cheng University, Chiayi 62102, Taiwan, Republic of China

**Keywords:** Gaussian process, genotype-phenotype map, fitness landscape

## Abstract

Multiplex assays of variant effect (MAVEs) allow the functional characterization of an unprecedented number of sequence variants in both gene regulatory regions and protein coding sequences. This has enabled the study of nearly complete combinatorial libraries of mutational variants and revealed the widespread influence of higher-order genetic interactions that arise when multiple mutations are combined. However, the lack of appropriate tools for exploratory analysis of this high-dimensional data limits our overall understanding of the main qualitative properties of complex genotype-phenotype maps. To fill this gap, we have developed *gpmap-tools* (https://github.com/cmarti/gpmap-tools), a *python* library that integrates Gaussian process models for inference, phenotypic imputation, and error estimation from incomplete and noisy MAVE data and collections of natural sequences, together with methods for summarizing patterns of higher-order epistasis and non-linear dimensionality reduction techniques that allow visualization of genotype-phenotype maps containing up to millions of genotypes. Here, we used *gpmap-tools* to study the genotype-phenotype map of the Shine-Dalgarno sequence, a motif that modulates binding of the 16S rRNA to the 5’ untranslated region (UTR) of mRNAs through base pair complementarity during translation initiation in prokaryotes. We inferred full combinatorial landscapes containing 262,144 different sequences from the sequences of 5,311 5’UTRs in the *E. coli* genome and from experimental MAVE data. Visualizations of the inferred landscapes were largely consistent with each other, and unveiled a simple molecular mechanism underlying the highly epistatic genotype-phenotype map of the Shine-Dalgarno sequence.

## Introduction

The genotype-phenotype map is a fundamental concept in genetics and evolutionary biology, encapsulating the relationship between sequence variation and its phenotypic outcome. Understanding this relationship is crucial for evolution (1–8), human genetic and infections disease as well as cancer (9–11), and also for synthetic biology and protein engineering (12–14) and plant and animal breeding (15–18). This task is inherently challenging because the effects of mutations often depend on the genetic background in which they occur due to the presence of genetic interactions within and between genes (19–22).

One approach to study empirical genotype-phenotype maps is by constructing sequences of interest and evaluating their biological function experimentally. Historically, our limited ability to engineer large number of genetic variants and measure their phenotypes has constrained our knowledge to phenotypic landscapes containing only a small number of genotypes (23–26). However, Multiplex Assays of Variant Effects (MAVEs) have emerged as a powerful tool, enabling the simultaneous measurement of molecular phenotypes for vast libraries of genetic variants in a single experiment (27, 28). These techniques have allowed quantifying the biological functionality of a large fraction of possible sequences for short regulatory elements (29–36), at specific positions in RNAs (37–41) and proteins (21, 42–52), or for specific combinations of mutations across different genes (53). The highly combinatorial nature of this data makes interpretation challenging, often requiring specialized software powered by sophisticated latent variable models and neural networks to accurately fit the data and make phenotypic predictions (54–58). Another promising family of approaches for modeling complex sequence-function relationships are Gaussian process models (59). Following this line of work, recently developed Gaussian process models naturally incorporate genetic interactions of any order by specifying priors that control the type and magnitude of genetic interactions, and which allow inference of complete genotype-phenotype maps from MAVE data, achieving state-of-the-art predictive power (60, 61).

An alternative approach to study genotype-phenotype maps is through collections of natural sequences. Because natural selection often acts to preserve functionality, we can assume that the probability of observing a sequence in nature depends on how well it performs its function. Thus, the probability distribution from which sequences with a specific function are drawn can be viewed as a genotype-phenotype map in which the phenotype is the probability of observing a sequence. Independent site models, such as Position-Weight Matrices, learn this probability distribution over possible sequences by assuming that positions are independent (62), whereas pairwise interaction models, also known as Potts models (63–66), relax this strong assumption by allowing interactions between pairs of positions. These models have been very successful in predicting structural contacts in proteins (67), mutational effects (68), novel functional proteins (69) and specific regulatory sequences, such as the splice sites (70). A recently proposed bayesian nonparametric model further generalizes these models by defining a prior distribution controlling the magnitude of local epistatic coefficients and inferring the complete probability distribution over sequence spaces under that prior, opening up the opportunity to study genotype-phenotype maps containing high-order epistatic interactions from readily available collections of natural sequences (71).

Another important challenge is the interpretation of these complex genotype-phenotype maps, particularly when the number of possible genotypes is large. A way to develop an intuitive understanding of complex datasets is through data visualization tools. Various strategies to represent genotype-phenotype maps have been proposed. The genotype-phenotype map has often been represented as a topographic map, where genotypes are points in a 2D space and the height represents the phenotype (1, 72). This conceptual representation is very intuitive and has provided us with language to qualitatively describe genotype-phenotype maps e.g. fitness peaks, valleys or plateaus. However, the space of possible sequences is a discrete space, which is more realistically represented as a Hamming graph, in which nodes represent genotypes and edges represent single-point mutations between them. For genotype-phenotype maps containing few genotypes, it often suffices to embed this graph in a two dimensional space using the distance to a reference sequence and the phenotype as coordinates (1, 37, 41, 48, 73) or specific embeddings of the Hamming graph (74) or subgraph induced by the set of highly functional sequences (44). A different strategy is to construct a low-dimensional representation that reflects the evolutionary dynamics induced by the genotype-phenotype map of interest, for example by having the distances between genotypes represent the expected time to evolve between them for a population evolving under selection for high phenotypic values (75). This is a very useful property for visualizing genotype-phenotype maps, as sets of functional sequences that are inaccessible to each other (peaks) are represented far apart in the visualization, naturally displaying the key genetic interactions separating them (valleys). This technique has been effectively applied to understand the qualitative features of a number of genotype-phenotype maps (60, 61, 71, 76, 77) and the constraints imposed by the structure of the genetic code in protein evolution (78). However, its broader applicability has been limited by the lack of accessible software packages implementing it.

Here, we present *gpmap-tools*, a *python* library that provides an integrated and accessible interface to methods for inference and visualization of large complex genotype-phenotype maps. Among other improved features, *gpmap-tools* incorporates a new computational back-end in which large matrices are represented as linear operators, enabling efficient computation. This allows us to easily sample from the prior distribution to simulate genotype-phenotype maps with different types and amounts of epistasis and to do statistical analysis of specific features of the genotype-phenotype maps in the presence of missing data through computation of the posterior distribution of arbitrary linear combinations of phenotypes e.g. mutational effects and epistatic coefficients across genetic backgrounds. Moreover, using the Laplace approximation, *gpmap-tools*, allows these same calculations to be conducted when using non-Gaussian likelihood functions, such as when inferring genotype-phenotype maps from collections of natural sequences. We also present a new projection operator that enables calculation of the variance explained by interactions between sets of sites in a complete genotype-phenotype map, providing useful statistics to understand the patterns and complexity of genetic interactions across different positions. In addition, *gpmap-tools* provides an extended interface for visualizing genotype-phenotype maps with different number of alleles per site, new functionality to investigate the sequence features that characterize different regions of the representation, tools for interactive visualization, and accelerated rendering of plots containing up to millions of genotypes.

We demonstrate the capabilities of *gpmap-tools* by inferring the fitness landscape of the Shine-Dalgarno (SD) sequence from two different types of data: i) sequence diversity across the 5’ untranslated regions (UTRs) in bacterial genomes and ii) MAVE data (31). The inferred landscapes show a common structure consisting of peaks corresponding to the 16S rRNA binding at different distances relative to the start codon, with registers separated by 3 nucleotides forming extended ridges of functional sequences due to the quasi-repetitive nature of the canonical SD motif. Using this knowledge about the qualitative properties of the genotype-phentoype map, we fit a simplified mechanistic model whose parameters have clear biophysical interpretations, allowing us to disentangle the effects of mutations on binding at different registers in vivo, while recapitulating the observed main qualitative features.

## Approach

### Epistasis in genotype-phenotype maps.

A genotype-phenotype map is a function that assigns a phenotype, typically a scalar value, to every possible sequence of length ℓ on α alleles (where e.g. α=4 for DNA and α=20 for proteins). The genotype-phenotype map can be represented by an $\alpha^{\ell }$-dimensional vector f containing the phenotype for every genotype. *gpmap-tools* implements two different methods for measuring the amount and pattern of epistasis in a genotype-phenotype map, one based on the typical size of local epistatic interactions and the other based on the fraction of variance explained by different subsets of sites.

#### Local epistatic coefficients.

The traditional epistatic coefficient quantifies how much the effect of a mutation A→a in one site changes in the presence of an additional mutation B→b in an otherwise identical genetic background C:

(1) $$\epsilon =\left(f_{aBC}-f_{ABC}\right)-\left(f_{abC}-f_{AbC}\right).$$

The average squared epistatic coefficient $\bar{\epsilon^{2}}$ across all possible pairs of mutations and across all possible genetic backgrounds C provides a measure of the variability in mutational effects between neighboring genotypes across the whole genotype-phenotype map (60). This measure $\bar{\epsilon^{2}}$ can be computed efficiently as a positive semi-definite quadratic form $\bar{\epsilon^{2}}=\frac{1}{s}f^{T}\Delta^{\left(2\right)}f$, where s is the number of epistatic coefficients and $\Delta^{\left(2\right)}$ is a previously described sparse $\alpha^{\ell }\times \alpha^{\ell }$ matrix (60). This statistic can also be generalized to $\bar{{\epsilon_{P}}^{2}}=\frac{1}{s_{P}}f^{T}\Delta^{\left(P\right)}f$ for local epistatic coefficients of any order P to characterize the typical size of local P-way epistatic interactions in a mean square sense (71).

#### Variance components.

Any genotype-phenotype map f can be decomposed into the contribution of ℓ+1 orthogonal subspaces $f=\sum_{k}f_{k}$, where $f_{k}$ represent a function containing epistatic interactions solely of order k. These orthogonal components $f_{k}$ can be obtained by projecting the function f into the k-th order subspace using the known orthogonal projection matrix $P_{k}$ with entries given by the Krawtchauk polynomials and can be used to compute the relative contribution of the different orders of interactions to a genotype-phenotype map f (61,79–82). Moreover, here we show that each k-th order subspace can be further decomposed into ℓk smaller subspaces defined by the genetic interactions involving k specific sites. For instance, the 3rd order subspace can be broken down into the relative contributions of interactions among every possible combination of 3 sites. If we define U to be a subset of k sites and $P_{U}$ the projection matrix into the subspace defined by that subset of sites U, we can express the function as the sum of contributions of $2^{\ell }$ components $\left(f=\sum_{U}P_{U}f\right)$, where the $P_{U}$ are given by:

(2) $$P_{U}\left(x,y\right)=\alpha^{-\ell }\prod_{\begin{array}{c} p\in U\\ x_{p}=y_{p} \end{array}}\left(\alpha -1\right)\prod_{\begin{array}{c} p\in U\\ x_{p}\neq y_{p} \end{array}}\left(-1\right).$$

These projection matrices allow us to identify, not only the contribution of interactions of different order, but also which sites and subsets of sites are involved in those interactions, providing a finer-grained characterization of the sequence-function relationship. We can aggregate these components in different ways to compute other low dimensional summary statistics, such as the total variance explained by epistatic interactions of a specific order k, or across all orders k involving a site or a subset of sites (83, 84).

### Gaussian process inference of genotype-phenotype maps.

#### Interpretable priors.

Gaussian process models are a class of Bayesian models that place a multivariate Gaussian prior distribution over all possible functions and compute the posterior distribution given observed data (85). In our case, the prior distribution is a multivariate Gaussian distribution given by p(f)=N(0,K), typically characterized by its covariance matrix K or precision matrix C, where the covariance matrix K is most often defined through a kernel function $K\left(x_{i},x_{j}\right)$ that returns the prior covariance between any pair of sequences $x_{i},x_{j}$. Our aim is to define prior distributions for f with hyperparameters that have clear biological interpretations in terms of the type and extent of epistasis included in the prior. This not only allows us to better understand inference and prediction under each of these priors but also to set the hyperparameters of these priors in a principled way and to interpret their values when learned from data.

*gpmap-tools* implements two families of priors, one family that is defined in terms of local epistatic coefficients and a second that is defined in terms of variance components. The first prior parametrizes the prior distribution through its precision matrix $C=\frac{a}{s}\Delta^{\left(P\right)}$ assigning a prior probability to f depending on the average squared epistatic coefficient of order P, i.e. $log\;p(f)\propto -\frac{1}{2}f^{T}Cf$ (60, 71). This prior implicitly leaves genetic interactions of order k<P unconstrained, e.g. for P=2 additive effects are not penalized, and hence correspond to the use of an improper Gaussian prior. This family of priors has a single hyperparameter a that is inversely proportional to the expected squared local epistatic coefficient under the prior. As a→0, we assign the same prior probability to every possible genotype-phenotype map, so that the Maximum a Posteriori (MAP) matches exactly the Maximum likelihood estimate. On the other hand, as a→∞, we assign zero prior probability to genotype-phenotype maps with non-zero P-epistatic coefficients, which is equivalent to fitting a model with epistatic interactions up to order P-1 (71). The second family of priors are the variance component priors, which are parametrized by their covariance matrix $K=\sum_{k=0}^{\ell }\lambda_{k}K_{k}$. It has ℓ+1 hyperparameters $\lambda_{k}$ that control the variance explained by genetic interactions of order k (86) and which equivalently control the decay in the predictability of mutational effects and epistatic coefficients in genetic backgrounds separated by an increasing number of mutations (61). The formal relationship between the two sets of priors is that the priors based on the $\Delta^{\left(P\right)}$ operators can be obtained as limits of the variance component prior (61).

#### Posterior distributions.

Given observations y for some set of sequences x, we update the probability distribution of plausible genotype-phenotype maps to be consistent with these observations by computing the posterior distribution p(f∣y). Under a Gaussian likelihood with known sequence-specific variance for the measurement error $p\left(y|f_{x},D_{\sigma^{2}}\right)=N\left(f_{x},D_{\sigma^{2}}\right)$, where $D_{\sigma^{2}}$ is a diagonal matrix with the variance $\sigma_{x}^{2}$ associated to each measurement x along the diagonal, the posterior distribution for the phenotype f is also a multivariate Gaussian, whose mean we write as $\overset{ˆ}{f}$ and whose covariance matrix we write as Σ.

Our approach for calculating $\overset{ˆ}{f}$ and Σ differs depending on whether we are using the prior based on the mean squared local epistatic coefficient or our variance component prior. For the prior based on the mean squared local epistatic coefficient, $\overset{ˆ}{f}$ is given by the solution of:

(3) $$\left(C+XD_{\sigma^{2}}^{-1}X^{T}\right)\overset{ˆ}{f}=XD_{\sigma^{2}}^{-1}y$$

where X is a sparse matrix with $X_{ij}=1$ if sequence i is the j-th sequence in x and $X_{ij}=0$ otherwise. Σ is given by:

(4) $$\Sigma ={\left(C+XD_{\sigma^{2}}^{-1}X^{T}\right)}^{-1},$$

(for derivation, see Supplementary Information). This formulation is useful when the $\alpha^{\ell }\times \alpha^{\ell }$ precision or cost matrix C is sparse, as is the case here, since then Equation 3 can be solved numerically using iterative methods. For the variance component prior, since it is defined in terms of the covariance matrix K, we can use the classical solution for Gaussian processes (85):

(5) $$\overset{ˆ}{f}=KX\;{\left(X^{T}KX+D_{\sigma^{2}}\right)}^{-1}y$$

(6) $$\Sigma =K-KX\;{\left(X^{T}KX+D_{\sigma^{2}}\right)}^{-1}X^{T}K.\;$$

The above solutions are completely general, in the sense that they hold for arbitrary valid prior covariance of precision matrices. However, as we will explain below, *gpmap-tools* implements highly optimized versions of these calculations that take advantage of the structure of sequence space and our specific choices for C and K.

### Inference of genotype-phenotype maps with *gpmap–tools*.

*gpmap-tools* provides a number of methods to infer complete genotype-phenotype maps using either phenotypic measurements of specific genotypes (including the possible user-provided error estimates for each sequence) or from collections of known functional sequences even in the absence of direct phenotypic measurements.

#### Minimum epistasis interpolation / local epistatic coefficient priors.

The minimum epistasis interpolation method was originally proposed (60) in terms of finding the $f_{z}$ at unobserved sequences z given the known $f_{x}$ at sequences x by minimizing $\bar{\epsilon^{2}}$ over the complete genotype-phenotype map and *gpmap-tools* provides a slight generalization to local epistatic coefficients of any order P, (by minimizing $f^{T}\Delta^{\left(P\right)}f$). Through use of the local epistatic coefficient prior, *gpmap-tools* also allows Gaussian process regression under the improper prior with precision matrix C and the presence of Gaussian measurement error given by $D_{\sigma^{2}}$. Using this local epistatic coefficient prior, the value of the hyperparameter a controlling the magnitude of the local P-th order epistatic coefficients is optimized via cross-validation. In addition to the point estimate $\overset{ˆ}{f}$ of the reconstructed genotype-phenotype map, *gpmap-tools* can also provide uncertainty quantification via the posterior covariance given in Equation 4.

#### Empirical variance component regression.

Empirical variance component regression (VC regression), proposed in (61), combines a Variance Component prior parameterized by the variance $\lambda_{k}$ associated to interactions of every order k with a Gaussian likelihood with known noise variance $D_{\sigma^{2}}$ to compute the exact Gaussian posterior distribution over f using equations 5 and 6. The hyperparameters $\lambda_{k}$ controlling the action of the prior are optimized through kernel alignment, this is, by minimizing the squared distance between the covariance under the prior and the empirical distance-covariance function computed from the incomplete data. This can be done very efficiently, because the prior correlation between two sequences depends only on the Hamming distance, reducing it to a lower dimensional constrained weighted least squares problem (61).

#### Sequence probability distribution estimation.

*gpmap-tools* also implements the SeqDEFT method (71) for estimating probability distributions over sequence space. SeqDEFT aims to infer the probability distribution π from which natural sequences are drawn. This problem is similar to the previous models if we consider the $log\pi_{i}$ as a phenotype associated to sequence i. Data typically consists of the number of times $N_{i}$ a given sequence i was observed out of a total of $N_{T}=\sum_{i}N_{i}$ observations and can be naturally modeled by a multinomial distribution parametrized by the probability $\pi_{i}$ of observing every possible sequence i:

(7) $$p\left(N|\pi \right)=Multinomial\left(N,\pi \right).$$

SeqDEFT parametrizes $\pi_{i}=\frac{e^{-\phi_{i}}}{\sum_{j}e^{-\phi_{j}}}$ and defines an improper prior distribution over the latent phenotype ϕ that penalizes local epistatic coefficients of order $P\;log\;p(\phi )\propto -\frac{1}{2}\phi^{T}C\phi$. As there is no analytical solution for the posterior distribution under this non-Gaussian likelihood function

(8) $$\log\;p\left(\phi |N,a\right)\propto -\frac{a}{2s}\phi^{T}\Delta^{\left(P\right)}\phi -\sum_{i}N_{i}\phi_{i}-N_{T}\sum_{i}e^{-\phi_{i}},$$

we resort to optimization methods to obtain the Maximum a Posteriori (MAP) estimate $\overset{ˆ}{\phi }=arg\;{max\;}_{\phi }log\;p(\phi |N,a)$ given a fixed value of the hyperparameter a. We use cross-validation and one-dimensional grid search to characterize how the log-likelihood in held-out data changes as a function of a and select the optimal value $a^{*}$(71). In addition to computing the MAP $\overset{ˆ}{\phi }$ under $a^{*}$, *gpmap-tools* implements the Laplace approximation (85) to the posterior distribution of ϕ as a multivariate normal distribution with mean $\overset{ˆ}{\phi }$ and covariance matrix given by the inverse of the Hessian evaluated at the MAP $({\left(\nabla \nabla_{\phi }log\;p(\overset{ˆ}{\phi }|N,a)\right)}^{-1}={\left(C+D_{\overset{ˆ}{\phi }}\right)}^{-1}$, where $D_{\overset{ˆ}{\phi }}$ is the diagonal matrix with $N_{T}e^{-\overset{ˆ}{\phi }}$ down its main diagonal).

### Visualization of genotype-phenotype maps.

Genotype-phenotype maps are inherently high-dimensional objects, and thus difficult to visualize in an intuitive manner. *Gpmap-tools* implements a previously proposed strategy for visualizing fitness landscapes (75) that computes embedding coordinates for genotypes such that squared distances between pairs of genotypes in the low-dimensional representation approximate the expected times to evolve from one to another under selection for high phenotypic values. This layout highlights regions of sequence space containing highly functional genotypes that are nevertheless poorly accessible to each other e.g. fitness peaks separated by valleys, or sets of sequences where the intermediates are functional but the order of the intervening mutations is highly constrained.

#### Evolutionary model.

We assume a weak mutation model of evolution in haploid populations, such that mutations are always fixed or lost before a new mutation arises (60, 71, 75). Under this model, the evolutionary rate Q(i,j) from genotype i to j depends on the mutation rate M(i,j) (which we assume is taken from a time-reversible mutational model) and the probability of fixation relative to a neutral mutation (87, 88):

(9) $$Q(i,j)=\left\{\begin{array}{ll} M(i,j)\frac{S(i,j)}{1-e^{S(i,j)}} & \text{if}\;i\;\text{and}\;j\;\text{are}\;\text{neighbors}\\ -\sum_{k\neq i}^{Q(i,k)}\; & \text{if}\;i=j\\ 0 & \text{Otherwise}, \end{array}\right.$$

where S(i,j) is the scaled selection coefficient for mutation from i to j. We can assume that this scaled selection coefficient is proportional to the phenotypic differences between the two genotypes (S(i,j)=c(f(j)-f(i)), where the constant c can be interpreted as the scaled selection coefficient ($2N_{e}s$, for a Haploid Wright-Fisher population) associated to a phenotypic difference of 1. Unless specifically studying the role of mutational biases on evolution on empirical landscapes, we would typically assume that M(i,j)=1 for any i,j pair (i.e. measure time in units of the inverse mutation rate), and focus on the evolutionary dynamics induced by the structure of the genotype-phenotype map alone. This model assigns a low but non-zero probability of fixation to deleterious mutations and has a unique stationary distribution π(i) given by

(10) $$\pi \left(i\right)=\frac{\pi_{M}\left(i\right)e^{cf\left(i\right)}}{\sum_{j}\pi_{M}\left(j\right)e^{cf\left(j\right)}}.$$

where $\pi_{M}(i)$ are the time-reversible neutral stationary frequencies, which are uniform in absence of mutational biases. The stationary distribution can be used to select a reasonable value of c for our evolutionary process. When representing a probability distribution, such as one inferred using SeqDEFT, setting f(i)=logp(i) and c=1 will result in a stochastic process in which the stationary distribution exactly matches the estimated genotype probabilities, providing a very natural representation of the landscape. When inferring the genotype-phenotype map from MAVE data, c can be adjusted so that the mean phenotype under the stationary distribution aligns with realistic natural values e.g. the phenotype associated to a wild-type or reference sequence(s). Alternatively, a range of c values can be used to generate a family of visualizations for a single genotype-phenotype map to reflect the evolutionary impact of the genotype-phenotype map under different assumptions concerning the relative strengths of selection and drift.

#### Low-dimensional representation.

The right eigenvectors $r_{k}$ of Q associated to the largest eigenvalues $\lambda_{k}\left(\lambda_{1}=0>\lambda_{2}\geq \right.{\;\lambda }_{3}\geq \ldots )$ can be computed using iterative methods that leverage the sparse structure of Q. When appropriately normalized and re-scaled as $u_{k}=\frac{1}{\sqrt{-\lambda_{k}}}\frac{r_{k}}{r_{k}^{T}D_{\pi }r_{k}}$, the first few $r_{k}$ for k≥2 can be used as embedding coordinates, resulting in a low dimensional representation in which squared distances between genotypes optimally approximate the commute times i.e. the sum of hitting times H(i,j) from i to j and H(j,i) from j to i, thus separating sets of functional genotypes that are largely inaccessible to each other for a population evolving under selection for high phenotypic values:

(11) $$\sum_{k=2}{\left(u_{k}(i)-u_{k}(j)\right)}^{2}\approx H\left(i,j\right)+H\left(j,i\right).$$

The eigenvalues $\lambda_{k}$ represent the rates at which the associated eigenvectors become less relevant for predicting evolutionary outcomes with time. The associated relaxation times $-\frac{1}{\lambda_{k}}$ have units of expected number of substitutions and allow us to identify components that decay slower than expected under neutral evolution, where we note that if all mutations occur at rate 1, the neutral relaxation time is given by the reciprocal of the minimum number of alleles across sites. Because $u_{k}$ captures the k-1-th strongest barrier to the movement of a population in sequence space, we refer to $u_{k}$ as diffusion axis k-1.

#### Rendering and visualization.

In addition to computing the coordinates $u_{k}$, *gpmap-tools* provides functionality at both high and low levels to plot and render the visualizations of genotype-phenotype maps using different backend plotting libraries. This includes the standard plotting library in python, *matplotlib* (89), for generating highly customized visualizations, but also an equivalent interface to generate interactive 3D visualizations that display the sequence associated to each node of the graph by hovering the mouse over them using *plotly* (90). Moreover, as rendering large numbers of points and lines becomes limiting in large datasets, the *gpmap-tools* plotting library leverages the power of *datashader* (91) for efficiently rendering plots containing millions of different elements, achieving close to an order of magnitude speed up for large genotype-phenotype maps (Figure S1).

### Efficient computation with *gpmap-tools*.

We aim to study genotype-phenotype maps with a number of genotypes ranging from a few thousands up to millions. However, all of the described methods require computing with unreasonably large matrices of size $\alpha^{\ell }\times \alpha^{\ell }$ For instance, studying a genotype-phenotype map for 9 nucleotides, a naive implementation with need to build a 4^9^ × 4^9^ matrix requiring 512GB of memory using 64 bit floating point numbers and over 100 billion operations to compute matrix-vector products. While some of the matrices are sparse e.g. $\Delta^{\left(P\right)}$ and Q, allowing efficient storage and computation (60, 71, 75), other matrices e.g. $P_{U}$ and K, are dense.

*gpmap-tools* circumvents these challenges using two strategies. First, we note that every matrix A with entries $A_{ij}$ depending only on the Hamming distance between sequence i and j, such as $\Delta^{\left(P\right)}$ as well as the dense matrices $P_{k}$ and $K_{k}$, can be expressed as an ℓ-order polynomial in the Laplacian of the Hamming graph L (61). This enables efficient computation of matrix-vector products $Ab=\sum_{i}^{\ell }c_{i}L^{i}b$ by multiplying the vector b by L up to ℓ times e.g. $L^{2}b=L(Lb)$ and taking linear combinations of the results without explicitly building the possibly dense matrix A.

Second, we note that many of the relevant matrices can be obtained as ℓ-Kronecker products of α×α matrices, such as $P_{U}=\overset{\ell }{\underset{p}{⨂}}P_{p}$. By using *scipy*’s (92) LinearOperators functionality, we can leverage the mixed Kronecker matrix-vector product property to efficiently compute e.g. $P_{U}b$ without constructing $P_{U}$ (see Supplementary Information). Rather than calculating explicit inverse matrices, we can likewise use these linear operators to find numerical solutions to matrix equations using Conjugate Gradient (CG). By combining multiple linear operators, we are able to compute the posterior variance for a small number of sequences of interest or the posterior covariance for any set of linear combinations of phenotypic outcomes e.g. calculating posterior variance for mutational effects in specific genetic backgrounds and epistatic coefficients of any order, while limiting the number of linear systems to solve with CG to the number of linear combinations of interest.

## Results

In this section, we illustrate the power of *gpmap-tools* to study the genotype-phenotype map of the Shine-Dalgarno (SD) sequence. The SD sequence is a motif located in the 5’UTR of most prokaryotic mRNAs recognized by the 3’tail of the 16S rRNA through base pair complementarity with a region known as the anti Shine-Dalgarno (aSD) sequence, promoting translation initiation (93). Understanding how the SD sequence modulates protein translation in vivo is key for optimizing protein production (94). Previous studies used existing sequence diversity (95) and MAVE experiments (31, 32) to build models for this genotype-phenotype map. However, these models cannot account for high-order genetic interactions and provided limited understanding of the structure of the genotype-phenotype map. Thus, *gpmap-tools* offers a new opportunity to model and understand the patterns of genetic interactions and the main qualitative features that define this important regulatory sequence.

### Inferring the probability distribution of the Shine-Dalgarno sequence.

**Fig. 1.**
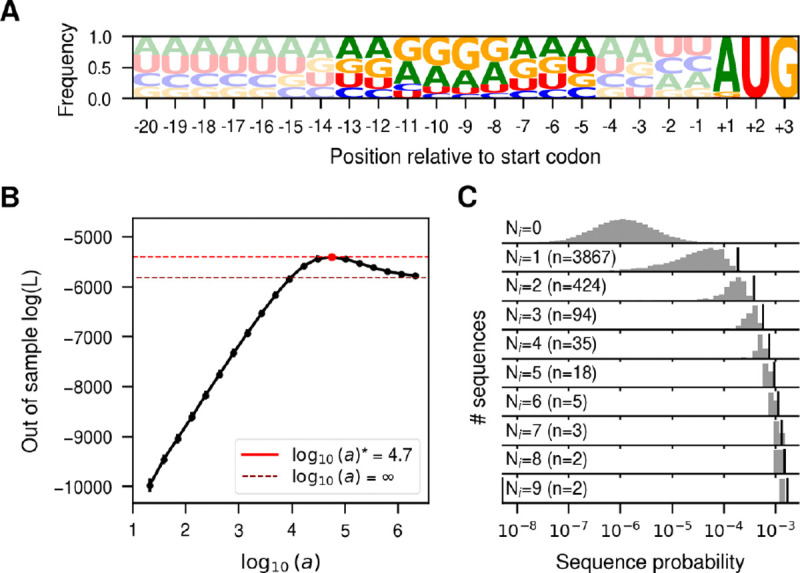
Inference of the probability distribution of the Shine-Dalgarno sequence. (A) Sequence logo representing the site-specific allele frequencies of 5,311 5’UTRs in the *E. coli* genome aligned with respect to the annotated start codon. The start codon and the 9 nucleotide region 4 bases upstream are highlighted to emphasize the region most relevant for translation initiation. (B) Log-likelihood computed in the 20% held-out sequences in 5-fold cross-validations of a series of SeqDEFT models (P=2) under varying values of the hyperparameter a. The horizontal dashed lines represent the log-likelihood of the limiting case maximum entropy model, corresponding to the independent sites model shown in panel A (black) or the best SeqDEFT model (red). (C) Distribution of inferred sequence probabilities depending on the number of times $N_{i}$ they were present in the *E. coli* genome represented in a logarithmic scale. Vertical black lines represent the empirical frequency $N_{i}/N_{T}$ corresponding to each $N_{i}$ value.

Here, we use SeqDEFT to infer the sequence probability distribution for the SD sequence by using the 5’untranslated regions (UTRs) across the whole *E. coli* genome. We extracted the 5’UTR sequence from 5,311 annotated genes and aligned them with respect to the start codon. Figure 1A shows site-specific allele frequencies for up to 20bp upstream of the start codon. This shows only a small bias towards increased G content between positions −13 and −5. While this observation suggests the location of the SD sequence relative to the start codon, the limited expressivity of this site-independent model is likely insufficient to capture the genotype-phenotype map underlying the sequence diversity of the SD sequence. Thus, we focused on the genotype-phenotype map of this 9 nucleotide region. Out of the total 4^9^ = 262, 144 possible sequences, we observe 3,690 unique sequences, most of them observed a single time. Given that the number of sampled sequences is two orders of magnitude smaller than the number of possible sequences, we expect many unobserved sequences to be functional and that sharing information across neighboring genotypes through SeqDEFT’s prior distribution could alleviate this limited amount of data. Figure 1B shows that the model predicts much better the frequencies of held-out sequences than either the site-independent model a=∞ or the empirical frequencies model a=0, providing strong support for the presence of epistatic interactions. We then computed the MAP solution (using all available data) under the value $a^{*}$ that maximized the likelihood for the held-out sequences and compared the inferred probabilities with the observed frequencies (Figure 1C). Sequences that appear more than 2–3 times in the genome are inferred to always be highly functional. However, there is a wide range of variability for unobserved sequences, ranging 4 orders of magnitude in their estimated probabilities, many of them with larger probabilities than some sequences that are observed once. The MAP shows a $\bar{\epsilon^{2}}=0.10$, corresponding to a root mean square local epistatic coefficient of 0.32, which is slightly less than half of the size of the root mean squared mutational effect (0.78). This indicates that making one mutation in the genetic background will often substantially change the effects of other mutations.

### Inferring the genotype-phenotype map of the Shine–Dalgarno sequence from MAVE.

We next use data from a previously published MAVE (31) measuring the expression of a GFP reporter controlled by a library of sequences containing nearly all 262,144 possible 9 nucleotide sequences 4 nucleotides upstream the start codon, as in our previous analysis. We first run MEI to predict the phenotype for all missing genotypes. The imputed genotype-phenotype map had an $\bar{\epsilon^{2}}=0.11$. While this value is not directly comparable with the results of our SeqDEFT analysis because of the difference in measurement scale (log probability vs. log GFP), we can again compare the root mean squared epistatic coefficient, which for MEI takes a value of 0.32, to the root mean squared size of mutational effects, which for MEI is 0.33, indicating that there is more epistasis in this dataset than inferred by our SeqDEFT analysis. Overall the relatively large amount of epistasis means that there is substantial variability in the effects of mutations across neighboring genotypes.

**Fig. 2.**
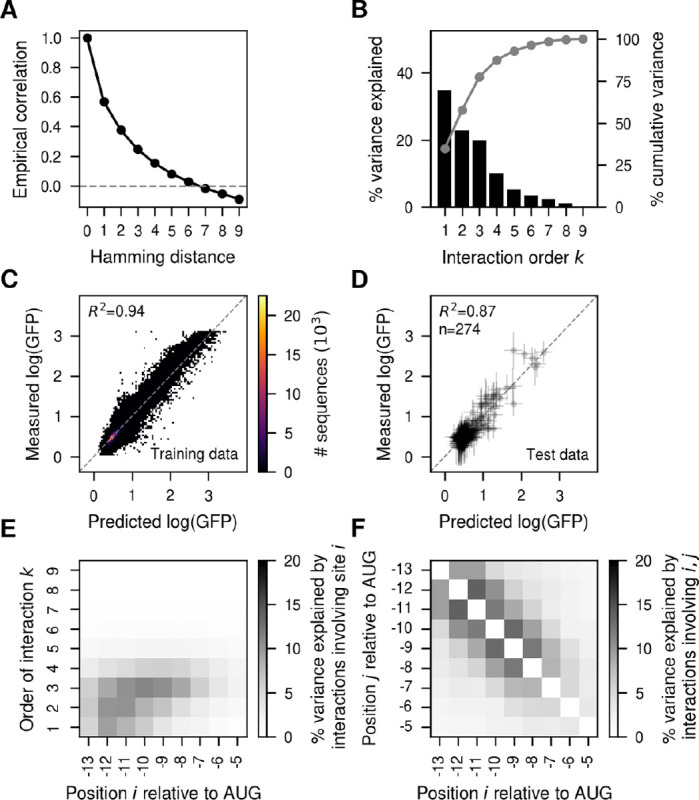
VC regression analysis of the experimentally measured genotype-phenotype map for the Shine-Dalgarno sequence in the dmsC gene context (31). (A) Empirical distance-correlation function using the measured log(GFP) values in the experimentally evaluated sequences. (B) Percentage of variance explained by interactions of order k in the inferred VC regression prior. Grey lines represent the cumulative percentage of variance explained by interactions up to order k. (C) Two-dimensional histogram showing the comparison of the measured log(GFP) and the MAP estimate under the VC model in sequences used for model fitting. (D) Comparison of the posterior distribution for held-out test sequences and the measured log(GFP) values. Horizontal error bars represent posterior uncertainty represented as the 95% credible interval, whereas vertical error bars correspond to the 95% confidence interval under each measurement’s variance. (E) Heatmap representing the percentage of variance explained by interactions of order k involving each position relative to the start codon. (F) Heatmap representing the percentage of variance explained by interactions of orders 2 or greater involving pairs of positions relative to the start codon.

To better capture this high degree of inferred epistasis, we turned to VC regression, where the prior reflects the observed predictability of mutational effects in the training data. We found that the empirical phenotypic correlation between pairs of sequences decayed quite quickly with the number of mutations e.g. pairs of sequences separated by three mutations only showed a correlation of 0.25 between their measured phenotypes (Figure 2A). We next estimated the variance component prior distribution that best matched the observed distance correlation patterns and computed the variance explained by interactions of every possible order under this prior (Figure 2B). The additive and pairwise component explained only 57.6% of the overall variance, suggesting an important influence of higher-order genetic interactions. We then inferred the complete genotype-phenotype map under this prior. These estimates recapitulated the experimental data extremely well ($R^{2}=0.94$, Figure 2C) and made predictions almost as accurate in held-out test sequences ($R^{2}=0.87$, Figure 2D). Importantly, our estimates of the uncertainty of the phenotypic predictions are well calibrated, as we find approximately the expected fraction of measurements in the test set within posterior credible intervals (Figure S2C). Comparing the predictive performance of MEI against VC regression as a function of the number of sequences used for training, we find that while the two models perform comparably when the genotype-phenotype map is densely sampled, and MEI performs better with extremely low sampling (likely due to error in the estimation of variance components), overall VC regression exhibited substantially higher performance across a wide range of training data densities (Figure S2A,B).

### Position-specific contributions to epistasis.

An important advance in *gpmap-tools* is its ability to use the $P_{U}$ matrices to evaluate the contribution of each site to genetic interactions of different order. Figure 2E shows this analysis for the MAP solution obtained using VC regression. We see that while positions −6 and −5 have an overall weak influence in the measured translational efficiency, sites −13 to −10 have both strong additive and epistatic contributions, whereas sites −9 to −7 influence the phenotype mostly through higher-order epistatic interactions. Thus, we find that sites in the SD sequence have very heterogenous contributions to genetic interactions of different orders, with some sites having stronger additive and lower order epistatic interactions, whereas other sites influence translation primarily via higher-order interactions. To investigate the presence of communities of interacting sites, we evaluated the variance explained by epistatic interactions of any order involving each possible pair of sites (Figure 2F). We found that all sites strongly interact with neighboring sites up to a distance of 4 nucleotides, a pattern that is compatible with communities of 4 consecutive sites across the 9 nucleotide sequence.

### Visualizing the probability distribution of the SD sequence.

**Fig. 3.**
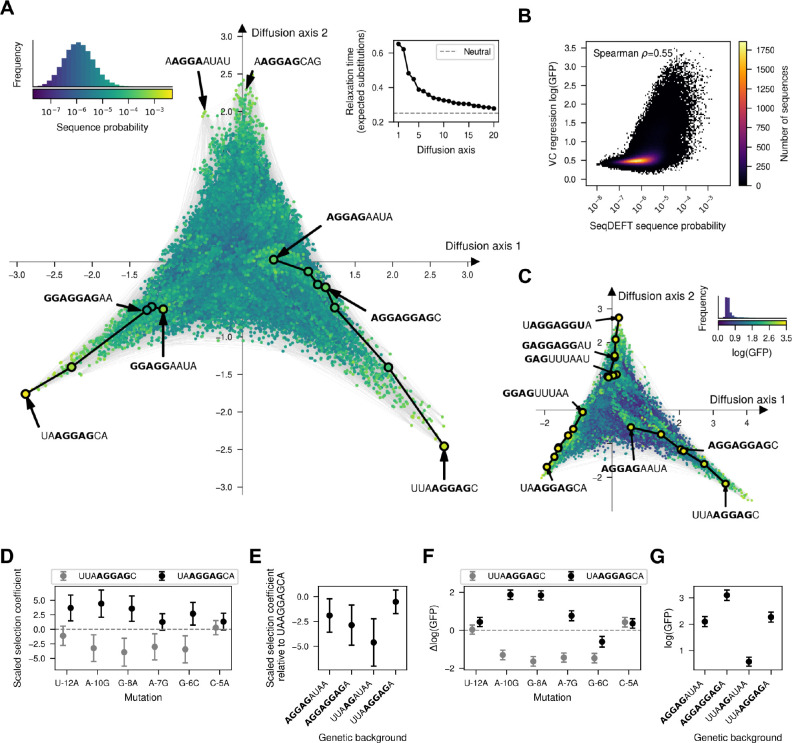
Visualization of the genotype-phenotype map of the Shine-Dalgarno sequence. (A,C) Low dimensional representation of the *E. coli* Shine-Dalgarno sequence probability distribution inferred with SeqDEFT (A) and the translational efficiencies inferred with VC regression (C). Every dot represents one of the possible 4^9^ possible sequences and is colored according to their inferred probability (A) or log(GFP) values (C). The inset represents the distribution of inferred sequence probabilities or log(GFP) values along with their corresponding color in the map. Inset in the upper right corner of (A) shows the relaxation times associated to the 20 most relevant Diffusion axes, showing that the first two Diffusion axes have much longer relaxation times than the rest. Sequences are laid out with coordinates given by these first two Diffusion axes and dots are plotted in the order by the 3rd Diffusion axis. (B) Two-dimensional histogram representing the relationship between the inferred sequence probabilities from their frequency in the *E. coli* genome and the estimated translational efficiencies inferred with VC regression from MAVE data. (D,F) Posterior distribution of the effects of specific mutations when introduced in two genetic contexts, UUAAGGAGC and UAAGGAGCA, which represent a shift in the position of the AGGAG motif. Posterior distributions from SeqDEFT (D) and VC regression models (F). (E,G) Posterior distribution of phenotypes associated to genotypes representing the shift of the AGGAG motif by 3 positions using SeqDEFT (E) or VC regression models (G). Points represent the Maximum a posteriori (MAP) estimates and error bars represent the 95% credible intervals.

In order to understand the main qualitative properties of this highly epistatic genotype-phenotype map, we generated a low dimensional representation using our visualization technique. Figure 3A shows that the genotype-phenotype maps consists of at least three largely isolated peaks. These peaks correspond to the canonical SD motif AGGAG located at three consecutive positions relative to the start codon, with a fourth central peak corresponding to a shift of the canonical motif one additional base upstream appearing along Diffusion axes 3 in a 3-dimensional representation (Figure S3). This shows that not only the aSD sequence can bind at different distances from the start codon to induce efficient translation initiation, consistent with the interaction neighborhoods shown in Figure 2D, but also that it is hard to evolve a sequence with a shifted SD motif by one or two positions through single point mutations without losing translational efficiency. In contrast, sequences with an SD motif shifted by three positions remain largely connected by extended ridges of functional sequences, in which a second binding site can evolve through a sequence of point mutations paths without destroying the first. Specifically, within each trinucleotide sequence around the central AGG common to the two binding registers, mutations can accumulate in diverse orders, opening up many different evolutionary paths only subject to the constraint of evolving a second SD motif before destroying the first one. Figure 3A highlights two examples of such paths.

### Comparing sequence probability across different species.

To investigate whether the structure of the genotype-phenotype map is the same across distant species, we performed the same analysis using 5’UTR sequences from 4,328 annotated genes in the genome of the distant *B. subtilis*. We first found that the AG bias marking the location of the SD sequence in the 5’UTR is located about 2 bp further upstream from the start codon compared to its location in *E. coli* (Figure S4A), as previously reported (95). We then extracted the 9 nucleotides sequences 6 bp upstream of the start codon and inferred the sequence probability distribution using SeqDEFT. The estimated log-probabilities were highly correlated with those obtained from the *E. coli* genome (Spearman r=0.94, Figure S4B), but more importantly, the inferred genotype-phenotype map displayed the same type of structure, with peaks corresponding to different binding registers of the aSD sequence and extended ridges connecting sets of sequences with overlapping binding sequences separated by 3 positions (Figure S4B). Overall, the probability distributions of the SD sequences are quantitatively very similar across distant species and shows the same main qualitative features.

### Comparing sequence probability and functional measurements.

We next compared the genotype-phenotype maps inferred based on observed genomic sequences with the genotype-phenotype map obtained with MAVE data. First, we directly compared the estimated sequence probability across the *E. coli* genome with the inferred translational efficiency from MAVE data (Figure 3B) for every possible sequence. We found a moderate non-linear relationship between these two independently inferred quantities (Spear man ρ=0.55). Sequences with very low estimated probability $\left(P<10^{-8}\right)$ consistently showed low translational efficiency (log⁡(GFP) < 1.0), whereas sequences with high sequence probability $\left(P>10^{-4}\right)$ had consistently higher but variable translational efficiencies (mean=1.84, standard deviation=0.63).

To investigate whether modest agreement is due to noise in the estimates for individual sequences or to having inferred qualitatively different genotype-phenotype maps, we applied the visualization technique to the empirical genotype-phenotype map inferred with VC regression (Figure 3C and S5). Despite the much more skewed phenotypic distribution of estimated translational efficiencies, this low dimensional representation has essentially the same structure with isolated peaks corresponding to different distances of the SD motif to the start codon and extended ridges connecting sequences with SD motifs shifted by 3 positions separated along several Diffusion axes (Figure 3C and S6). In addition to the previous structure, we identify an additional extended ridge of functional sequences with sequences starting by GAG. This subsequence, together with the upstream G from the fixed genetic context in which the experiment was performed, forms a functional binding site for the aSD sequence. In contrast, the probability distribution of SD sequences was inferred from genomic sequences with different flanking sequences in which sequences starting with GAG, on average, are not as functional. Thus, we can conclude that, despite showing only a moderate quantitative agreement, the two inference procedures using different types of data are able to recover genotype-phenotype maps with the same qualitative features and expected long-term evolutionary dynamics.

### Evaluating the confidence of genetic interactions and phenotypic predictions.

Visualizations of the inferred genotype-phenotype maps have enabled the identification of their main qualitative features and the potential genetic interactions underlying them, but they rely on a point estimate of the genotype-phenotype map that does not take into account uncertainty. However, we can complement these analyses by leveraging the uncertainty quantification capabilities of our Gaussian process models as implemented in *gpmap-tools*. For example, we can compute the posterior distribution of the effects of specific mutations in different backgrounds in order to evaluate the strength of evidence in the data supporting different hypotheses suggested by the visualizations of MAP estimates. As an illustration of this strategy, we first validated the incompatibilities separating peaks by computing the posterior distribution for mutational effects in the two backgrounds UUA**A****G****GAG**C and UA**A****G****G****AG**CU, which contain the same AGGAG motif shifted by one position (Figure 3D). Mutations affecting sites outside the SD motifs in the two registers e.g. U-12A and C-5A, showed small and similar effects in the two genetic backgrounds (Figure 3D). In contrast, the three mutations that allow shifting the SD motif one position upstream (A-10G, G-8A, A-7G) have strong effects with opposite signs in the two genetic contexts (Figure 3D). Importantly, the posterior distributions are concentrated around the means, showing that the data strongly supports that mutations needed to shift the SD motif by one position are substantially deleterious in that context, creating the valleys that separate the main peaks of this genotype-phenotype map.

We next evaluated the evidence supporting the existence of the extended ridge of functional sequences that shifts the SD motif by 3 positions. To do so, we computed the posterior distribution at four specific genotypes that contain the two binding registers, only one, or none. Whereas **AGGAGG**UAA, UAA**AGGAGG** and **AGGAGGAGG** are highly functional sequences, as they allow binding of the aSD sequence at either or both positions, if the first three nucleotides are mutated first as in UUA**AGG**UAA the first SD motif is destroyed before evolving the second one, resulting in low translational efficiency (Figure 3E). The posteriors for these same genotypes and mutations from the *B. subtilis* genome (Figure S4D,E) and from VC regression analysis on MAVE data (Figure 3F,G) are largely concordant and allow us to conclude that the evidence for this particular high fitness ridge is stronger from the MAVE data than it is from the *E. coli* or *B. subtilis* genomic sequence data.

### A biophysical model recapitulates the qualitative properties of empirical SD genotype-phenotype maps.

Despite inferring a highly epistatic genotype-phenotype map from the experimental data, the visualization revealed that it can be explained by a rather simple underlying mechanism consisting on the ability of the aSD sequence to bind at different distances from the start codon. We hypothesize that this mechanism alone explains both the existence of isolated peaks and, together with the quasi-repetive nature of the aSD sequence, the extended ridges. Moreover, despite our ability to estimate mutational effects in different contexts, inference of the actual binding preferences of the aSD from the data is hindered by the convolution of the effects of mutations on the binding at different registers. To tackle these issues, we fit a simple mechanistic model, in which the measured protein abundance is linearly dependent on the fraction of mRNA bound by the aSD at thermodynamic equilibrium at different positions p relative to the start codon, where the binding energy ΔG of the aSD is an additive function of the sequence at that position $x_{p}$ (see Methods).

**Fig. 4.**
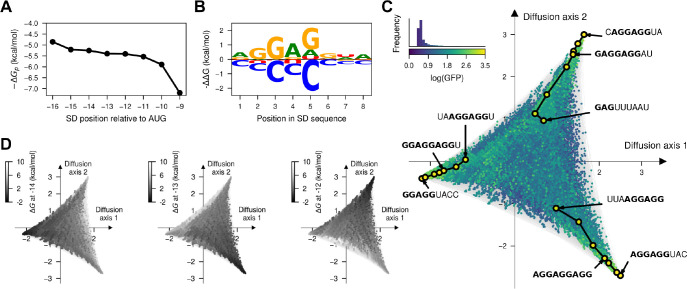
Thermodynamic model of sequence-dependent translational efficiency. (A) Estimated average free energy of binding at position p relative to the start codon across all possible sequences. (B) Sequence logo representing the site-specific but register-independent allelic contributions to the binding energy, where the size of the letter represents the difference in binding energy to the average across nucleotides. (C) Visualization of the genotype-phenotype map that results from predicting the phenotype of every possible sequence under the inferred thermodynamic model. Every dot represents one of the possible 4^9^ possible sequences and is colored according to the predicted log⁡(GFP). The inset represents the phenotypic distribution along with their corresponding color in the map. Sequences are laid out according to the first two Diffusion axes and dots are plotted in order according to the predicted log⁡(GFP). (D) Visualization of the genotype-phenotype map under the inferred thermodynamic model representing the binding energies at positions −14, −13 and −12 relative to the start codon showing that the peaks in the visualization correspond to the strongest binding at different positions. Binding energies are reported in units of kcal/mol assuming a temperature of 37°C. Dots are plotted in reverse order of binding energy in the corresponding register.

We fit this biophysical model by maximum likelihood (Figure S7A) to the MAVE dataset and achieved good predictive performance in both training ($R^{2}=0.59$, Figure S7B) and held-out sequences ($R^{2}=0.64$, Figure S7C). Importantly, this model contains only 34 parameters that all have clear biophysical interpretations e.g. in terms of mutational effects on binding energies. The model also includes a parameter α that specifies the background fluorescence in absence of aSD binding to the 5’UTR, which we estimated as $\overset{ˆ}{\alpha }=0.47$. Likewise, the maximal protein abundance, obtained when the mRNA is saturated with aSD, is estimated to be 3.85. These estimates suggest that the equilibrium occupancy of mRNA’s 5’UTR by aSD is low for most sequences, since the dataset has a median protein abundance of 0.511 and a maximum of 3.12. Moreover, position specific binding energies $\Delta G_{p}$ are lower (more stable) further from the start codon (Figure 4A). This can be explained by either SD:aSD complex being stabilized by other trans elements binding further from the start codon, or by the aSD hindering the binding of Met-tRNA:AUG when located too close to each other. Moreover, we were able to deconvolve the effects of mutations on binding at different registers and inferred allele and position specific energetic contributions to binding (Figure 4B). As expected, the reverse complement of the aSD is the most stable binder, but different mutations have substantially variable effects in the binding energy. Not only do some positions have stronger energetic contributions in general (positions 2–5 within the SD sequence), but different missmatches with the aSD in the same position have different energetic effects e.g. A4G is only slightly destabilizing (ΔΔG=0.79kcal/mol), whereas A4C is highly destabilizing (ΔΔG=1.77kcal/mol). Importantly, predictions of this simple model for all 4^9^ SD sequences recapitulate the main structure of the genotype-phenotype map with isolated peaks and extended ridges corresponding to different registers of binding, as expected (Figure 4C). We can verify that the peaks correspond to different binding registers by computing the binding energy of every sequence at specific positions relative to the start codon and color the visualization by those energies (Figure 4D). Overall, the visualization allowed us to develop a simplified biophysically interpretable model and to verify that this model recapitulates the main qualitative features of the genotype-phenotype map of the Shine-Dalgarno sequence.

## Discussion

In this paper, we present *gpmap-tools*, an extensively documented software library with tools for the inference, visualization and interpretation of empirical genotype-phenotype maps containing arbitrarily complex higher-order genetic interactions. By providing a framework for the analysis of complex genetic interactions, *gpmap-tools* has the potential to reveal the simple qualitative properties of these complex mappings, and to aid in development of biophysical and mechanistic hypotheses for these observed features.

The first step in this framework is the inference of the complete genotype-phenotype comprising all possible sequences from either experimental MAVE data or sequence counts. Taking into consideration the noise in the data (due either to sampling noise or experimental error), *gpmap-tools* is capable of computing the high-dimensional posterior distribution over all possible genotype-phenotype maps under a variety of priors. This allows us to obtain the maximum a posteriori (MAP) estimate, this is, the most probable genotype-phenotype map given the observed data. However, in contrast to other expressive models able to capture complex genetic interactions e.g. neural networks (57, 96), our inference methods provide a rigorous quantification of the uncertainty about the phenotypes of specific sequences, mutational effects across genetic backgrounds and, more generally, any linear combination of the phenotypes of a number of genotypes. This is important, as it tells the user which phenotypic predictions, mutational effects or genetic interactions can be trusted and to what extent, given the information provided by the data.

*gpmap-tools* re-implements a powerful method for visualizing fitness landscapes (75) that allows exploratory data analysis, interpretation and comparison of these complex datasets. Thus, rather than interpreting the results through an explicit parametric model allowing high-order genetic interactions or descriptive statistics like the number of peaks or adaptive walks (26, 35, 97, 98), this method leverages the evolutionary dynamics on the genotype-phenotype map to highlight its main, potentially unexpected, qualitative features. Moreover, we can use it to generate hypotheses for how mutational effects change with genetic backgrounds, which can then be evaluated through the posterior distribution (Figure 3 and S4). Identifying the main features of the genotype-phenotype map can be crucial for defining an appropriate mechanistic or biophysical model. For instance, visualization of the Shine-Dalgarno genotype-phenotype map allowed us to define a thermodynamic model in which the binding energy depended only additively on the sequence at each register, while recapitulating the peaks observed in the data. Additionally, this technique enabled a detailed comparison of genotype-phenotype maps inferred with different methods and data sources and the extent to which they had the same structure, in contrast to broadly used metrics, like Pearson or Spearman correlation coefficients. In this work, we used it to show genotype-phenotype maps with essentially the same structure inferred from data from distant species like *E. coli* and *B. subtilis* and completely independent data sources (experimental MAVE data and observations of natural sequences). Finding consistent structures across different data types and sources is particularly relevant for understanding the role of the fitness landscape on evolution of these regulatory sequences because we do not have access to the true fitness values. More generally, the visualization technique opens up the opportunity to compare genotype-phenotype maps of different genetic elements e.g. regulatory sequences, protein-protein interactions, and enzymes, by for example finding shared fitness landscape structures that induce similar evolutionary dynamics despite the differing biological substrates.

*gpmap-tools* enables inference and interpretation of complex genotype-phenotype maps comprising millions of sequences by making a number of assumptions that introduce some limitations. First, MEM and VC regression are phenomenological models that do not explicitly account for non-specific epistasis e.g. biophysical models. While these models can still make highly accurate phenotypic predictions in the presence of global epistasis through pervasive specific interactions, this limits our ability to distinguish specific from non-specific genetic interactions (54–56, 99). Second, SeqDEFT assumes that observed sequences are drawn independently from the underlying probability distribution. While this assumption may hold for a few specific regulatory sequences that are repeated many times along the genome of a single species e.g. the Shine-Dalgarno sequence or the 5´ splice site (71), it remains unclear how robust it is to the known challenge of using phylogenetically related sequences from widespread multiple sequence alignments of protein families (100–102). Third, both inference and visualization methods still require storing all possible sequences and their phenotypes in memory, whose number grow exponentially with sequence length, thus limiting the applicability of *gpmap-tools* to spaces of sequences of a constant and relatively short length. Despite these limitations, *gpmap-tools* provides a unique set of tools for studying the structure of short sequence genotype-phenotype maps at an unprecedented scale, which is a necessary stepping stone towards understanding genotype-phenotype maps at the gene, protein or genome-wide scale.

## Methods

### Sequence diversity of the Shine-Dalgarno sequence.

We downloaded the *E. coli* genome and annotation from Ensembl bacteria release 51, built on the assembly version ASM160652v1, and *B. subtilis* assembly ASM904v1 from GeneBank. We extrated the 5’UTR sequence for every annotated gene using *pysam* (103, 104) and kept the 5,311 and 4,328 sequences, respectively, for which we could extract 20 bp upstream of the start codon without any ambiguous character ‘N’. These sequences were aligned with respect to the start codon and used for computing site-frequency logos using *logomaker* (105) and estimating the complex probability distribution using *gpmap-tools* implementation of SeqDEFT (71). The MAP estimate was used to compute the coordinates of a low dimensional representation assuming that the stationary distribution of the evolutionary random walk matches the estimated sequence probabilities for selecting a proportionality constant of c=1 and uniform mutation rates.

### Analysis of the experimental fitness landscape of the Shine-Dalgarno sequence.

Phenotype data was computed from the processed data for independent replicates conducted in the dmsC genetic background as reported in the original manuscript (31). The mean and standard error was computed for all the 257,565 measured sequences. We estimated a common measurement variance of ${\overset{ˆ}{\sigma }}^{2}=0.058$ using genotypes measured across all 3 experimental replicates. The squared standard error for each genotype i was computed by dividing the overall experimental variance ${\overset{ˆ}{\sigma }}^{2}$ by the number of replicates $n_{i}$ in which each sequence was measured $\left({\overset{ˆ}{\sigma }}_{i}^{2}={\overset{ˆ}{\sigma }}^{2}/n_{i}\right)$. We kept 0.1% of the sequences as test set, and use the remaining sequences for fitting different models to infer the complete genotype-phenotype map while evaluating their performance on the held-out test data. We estimated the variance components from the empirical distance-correlation function and used them to define a Gaussian process prior for inference of the complete combinatorial landscape containing all 4^9^ genotypes, taking into account the known experimental variance ${\overset{ˆ}{\sigma }}_{i}^{2}$ for each sequence. We also computed the posterior mean and variances across all test sequences to assess the accuracy of the predictions and the calibration of the posterior probabilities in held-out data. We used the MAP estimate to compute the coordinates of the visualization assuming several different average values of log(GFP) under the stationary distribution that ranged from 1 to 2.5 (Figure S5). An average log⁡(GFP) of 2 at stationarity was selected and used for all subsequent visualizations, similar to our best estimate of 2.03 for the wild-type reference.

### Thermodynamic model of the Shine-Dalgarno genotype-phenotype map.

We assume that translation is limited by the initiation step, which is itself modulated by the binding of the 16S rRNA to the 5’UTR of the mRNA, whose concentration is assumed to be independent of the Shine-Dalgarno sequence. Binding and dissociation are assumed to be much faster than the rate at which translation is effectively initiated, so that the protein abundance is proportional to the fraction of mRNA bound by the 16S rRNA in any configuration or register p at thermodynamic equilibrium. This quantity depends on the binding energy ΔG of the 16 S rRNA to the mRNA to the sequence $x_{p}$ located at position p, the temperature, which is assumed to the 37°C (310K), and the universal gas constant $R=1.9872\times 10^{-3\;}kcal/mol{\;K}^{-1}$. Moreover, we assume that there is a minimal value *α* that is independent of the variable 5’UTR sequence in the experiment, which can represent a background translation level that depends on a different mechanism of initiation, or background signal in the assay e.g. cells auto-fluorescence in the GFP channel. Thus, the overall protein abundance f(x) for a sequence x depends on α and the fraction of bound mRNA multiplied by the translation rate when bound β, which also encodes for the maximal protein output through this initiation mechanism:

(12) $$f\left(x\right)=\alpha +\beta \left(\frac{\sum_{p}\;e^{-\frac{\Delta G_{p}\left(x_{p}\right)}{RT}}}{1\;+\;\sum_{p}\;e^{-\frac{\Delta G_{p}\left(x_{p}\right)}{RT}}}\right).$$

We next assumed that the binding energy at position p depends only on the 8 nucleotide subsequence at that position, such that each position has an intrinsic preference of binding $\Delta G_{p}^{0}$ and a site independent contribution of the allele c at each position i of the sequence $x_{p}(i,c)$. Importantly, we extended the variable 9 nucleotide sequences with the fixed upstream and downstream sequences CCG and UGAG from the dmsC genetic context to incorporate the effect of mutations in binding registers spanning both fixed and variable regions of the sequence:

(13) $$\Delta G\left(x_{p}\right)=\Delta G_{p}^{0}+\sum_{i}\sum_{c}x_{p}\left(i,c\right)\Delta \Delta G_{ic}.$$

Finally, we assume that the measurement y for sequence x is observed with known noise variance $\sigma^{2}$ and an extra or uncharacterized variance $\tau^{2}$ under a Gaussian likelihood function given by $p(y|x)=N\left(f(x),\sigma^{2}+\tau^{2}\right)$. We used PyTorch to encode the model and the Adam optimizer with a learning rate of 0.01 for 2500 iterations, while monitoring for convergence (Figure S7A), to find the maximum likelihood estimates of the model parameters.

## Supplementary Material

Supplement 1
