# Supplementary material for: Inference and visualization of complex genotype-phenotype maps with gpmap-tools
Source: bioRxiv. 2025 Aug 26:2025.03.09.642267. Preprint. [Version 3] doi: 10.1101/2025.03.09.642267 (PMC11952336; doi:10.1101/2025.03.09.642267)
Supplement: 1 [file NIHPP2025.03.09.642267v3-supplement-1.pdf]

bioRxiv preprint doi: https://doi.org/10.1101/2025.03.09.642267; this version posted August 26, 2025. The copyright holder for this preprint (which was not certified by peer review) is the author/funder, who has granted bioRxiv a license to display the preprint in perpetuity. It is made available under aCC-BY 4.0 International license.

Supplementary Figures

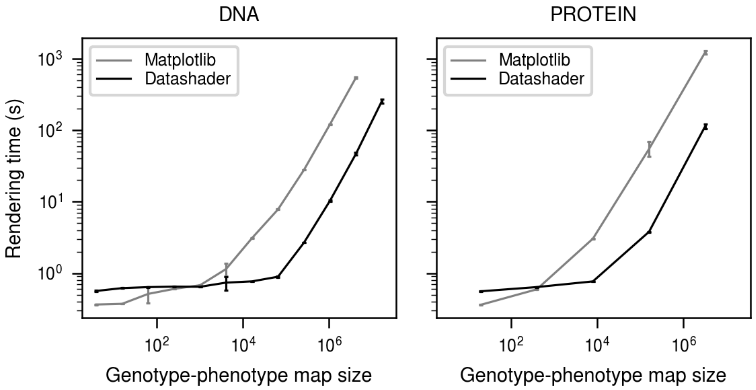

Fig. S1. Visualization rendering times using two different back-end libraries for plotting as a function of the size of DNA and protein genotype-phenotype maps.

bioRxiv preprint doi: https://doi.org/10.1101/2025.03.09.642267; this version posted August 26, 2025. The copyright holder for this preprint (which was not certified by peer review) is the author/funder, who has granted bioRxiv a license to display the preprint in perpetuity. It is made available under aCC-BY 4.0 International license.

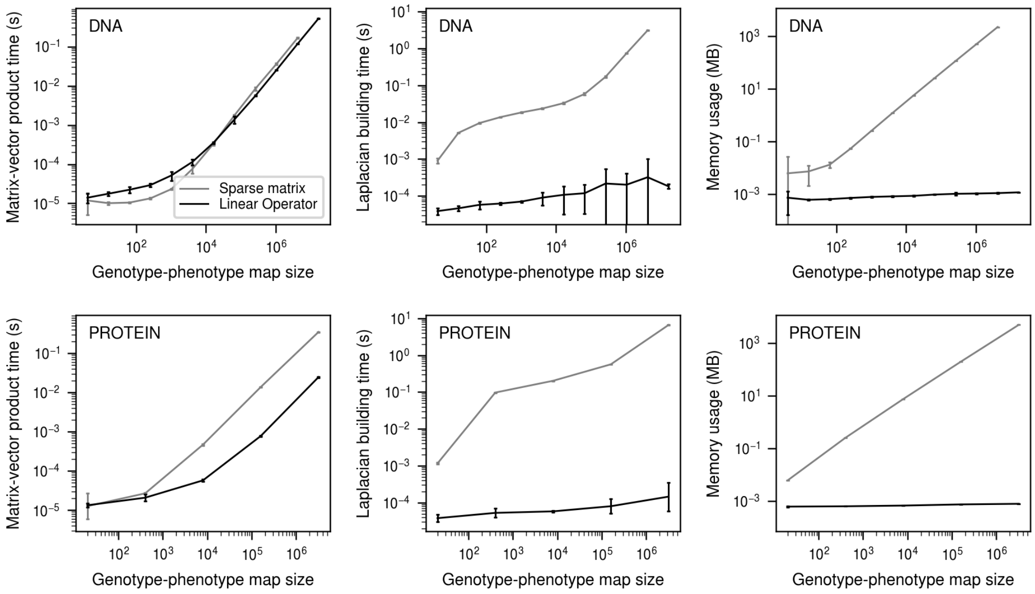

Fig. S2. Comparison of the running times and memory requirements for computation of matrix-vector products with the Laplacian of the Hamming graph using our new Linear Operator or our previous sparse matrix formulation.

bioRxiv preprint doi: https://doi.org/10.1101/2025.03.09.642267; this version posted August 26, 2025. The copyright holder for this preprint (which was not certified by peer review) is the author/funder, who has granted bioRxiv a license to display the preprint in perpetuity. It is made available under aCC-BY 4.0 International license.

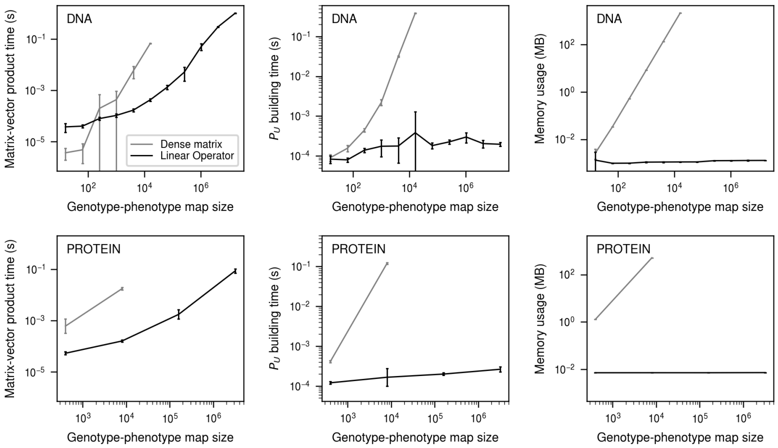

Fig. S3. Comparison of the running times and memory requirements for computation of P_U matrix-vector products using our Linear Operator or the corresponding dense matrix.

bioRxiv preprint doi: https://doi.org/10.1101/2025.03.09.642267; this version posted August 26, 2025. The copyright holder for this preprint (which was not certified by peer review) is the author/funder, who has granted bioRxiv a license to display the preprint in perpetuity. It is made available under aCC-BY 4.0 International license.

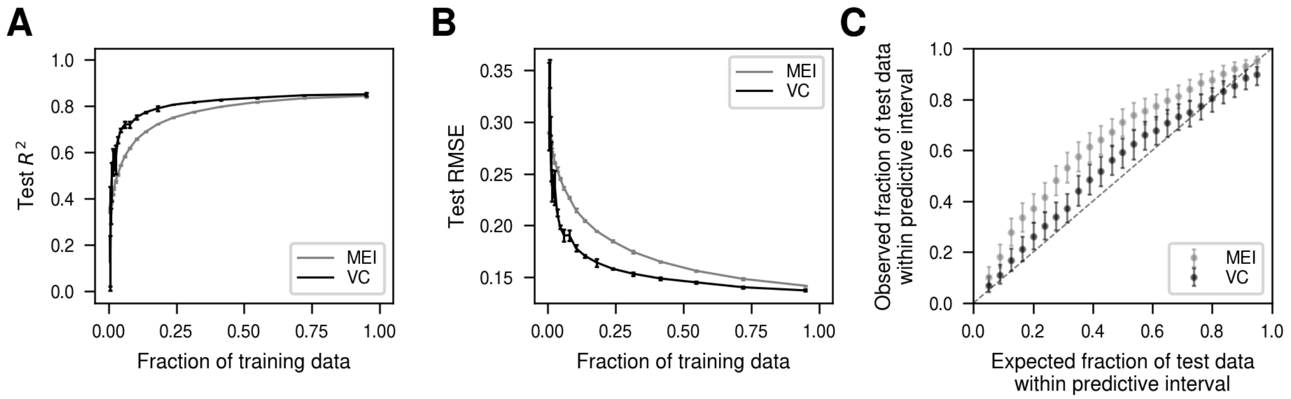

Fig. S4. Predictive performance of Minimum Epistasis Interpolation (MEI) and Variance Component (VC) regression in held-out data. (A,B) Model predictive performance measured by the R^2 (A) and RMSE (B) in held-out data as a function of the fraction of data used for training and phenotypic prediction. Error bars represent the standard deviation over 3 independent subsets of sequences used for training at each proportion. (C) Evaluation of the models calibration by comparing the expected fraction of times a predictive interval will contain the real phenotypic value compared to the fraction of times it actually contained the measured phenotype across 274 test data points. Error bars represent the 95% Jeffreys confidence interval for the estimated fraction of data points laying within the corresponding predictive interval. Diagonal dashed gray line shows the expectation under perfect model calibration.

bioRxiv preprint doi: https://doi.org/10.1101/2025.03.09.642267; this version posted August 26, 2025. The copyright holder for this preprint (which was not certified by peer review) is the author/funder, who has granted bioRxiv a license to display the preprint in perpetuity. It is made available under aCC-BY 4.0 International license.

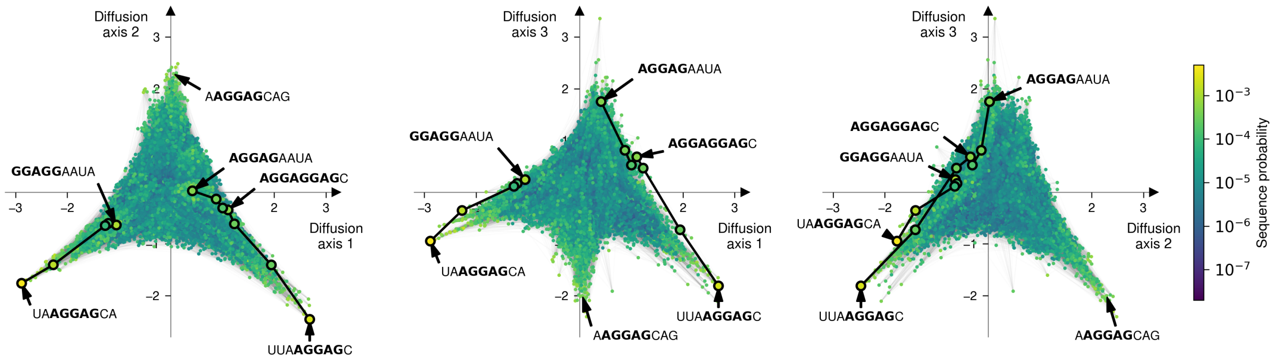

Fig. S5. Low-dimensional representation of the Shine-Dalgarno probability distribution inferred with SeqDEFT along Diffusion axes 1, 2 and 3. Every dot represents one of the possible 4^9 possible sequences and is colored according to their inferred probability. Sequences are laid out according to the indicated Diffusion axes and dots are plotted in order according to the missing Diffusion axis.

bioRxiv preprint doi: https://doi.org/10.1101/2025.03.09.642267; this version posted August 26, 2025. The copyright holder for this preprint (which was not certified by peer review) is the author/funder, who has granted bioRxiv a license to display the preprint in perpetuity. It is made available under aCC-BY 4.0 International license.

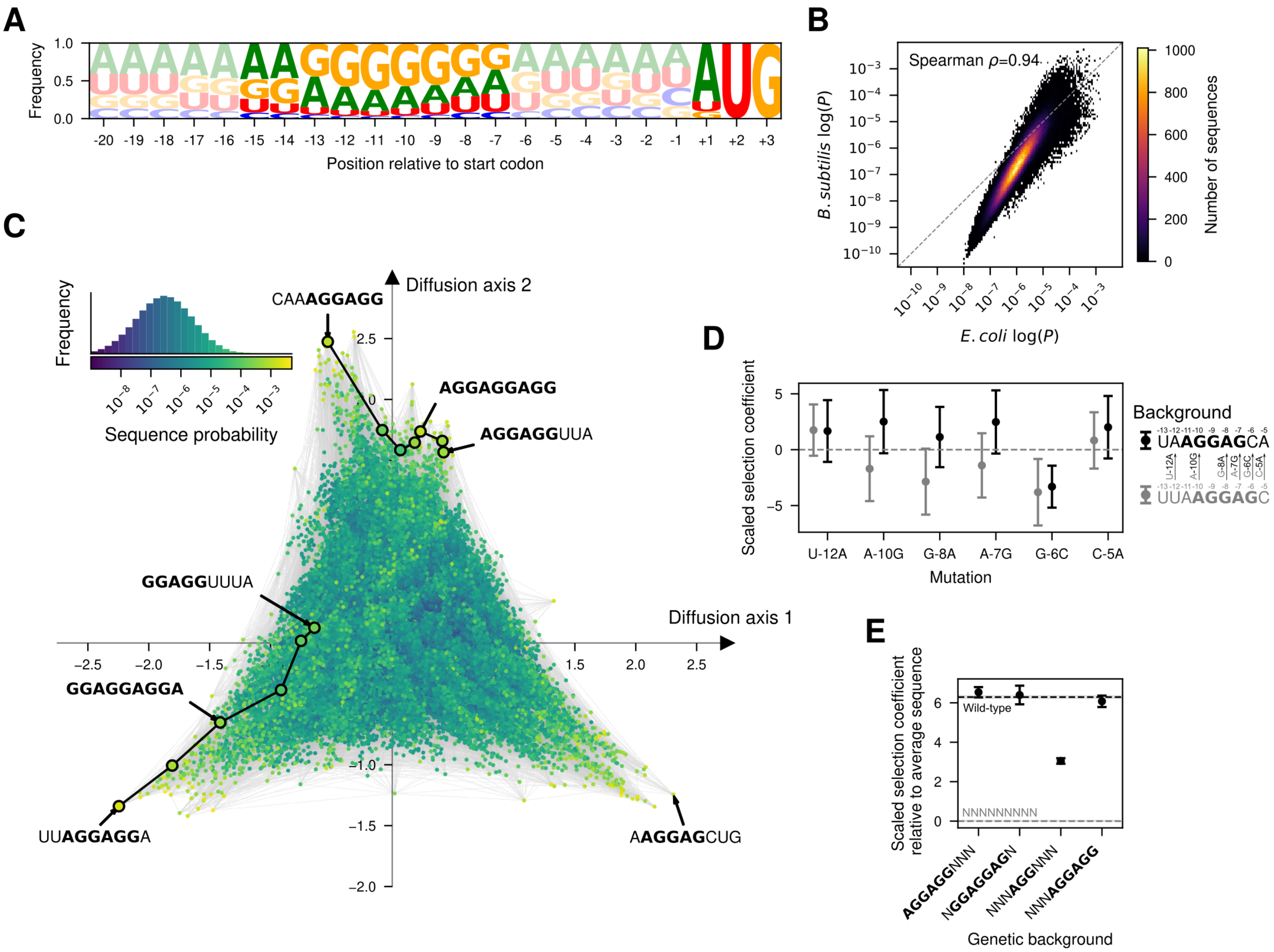

Fig. S6. The structure of the genotype-phenotype map inferred from *B. subtilis* is conserved. (A) Sequence logo representing the site-specific allele frequencies of 4,328 5'UTRs in the *B. subtilis* genome aligned with respect to the annotated start codon. The start codon and the 9 nucleotide sequences 6 bases upstream are highlighted to emphasize the most relevant cis-regulatory sequences for translation initiation. (B) Two-dimensional histogram representing the relationship between the inferred sequence probabilities from their frequency in the *E. coli* and *B. subtilis* genomes. (C) Low-dimensional representation of the Shine-Dalgarno probability distribution inferred with SeqDEFT. Every dot represents one of the possible 4^9 possible sequences and is colored according to its inferred probability. The inset represents the distribution of inferred sequence probabilities along with their corresponding color in the visualization. Sequences are laid out according to the first two Diffusion axes and dots are plotted in order according to the 3rd Diffusion axis. (D) Posterior distribution inferred by SeqDEFT for the scaled selection coefficient of specific mutations when introduced in two genetic contexts, UUAAGGAGC and UAAGGAGCA, representing a shift of the AGGAG motif by one nucleotide. Note that estimated mutational effects, except for G-6C, are largely compatible with those estimated from the *E. coli* genome shown in Figure 4D in the UAAGGAGCA context. (E) Posterior distribution inferred by SeqDEFT for the average scaled selection coefficient, relative to the average across all possible sequences, for genotypes containing the AGGAGG motif at positions separated by three nucleotides, along with their potential mutational intermediates. Horizontal dashed lines represent posterior mean of the average phenotype across all possible sequences (grey) or wild-type genomic sequences (black). Shaded areas represent the 95% credible intervals. (D,E) Points represent the maximum a posteriori (MAP) estimates and error bars represent the 95% credible intervals.

bioRxiv preprint doi: https://doi.org/10.1101/2025.03.09.642267; this version posted August 26, 2025. The copyright holder for this preprint (which was not certified by peer review) is the author/funder, who has granted bioRxiv a license to display the preprint in perpetuity. It is made available under aCC-BY 4.0 International license.

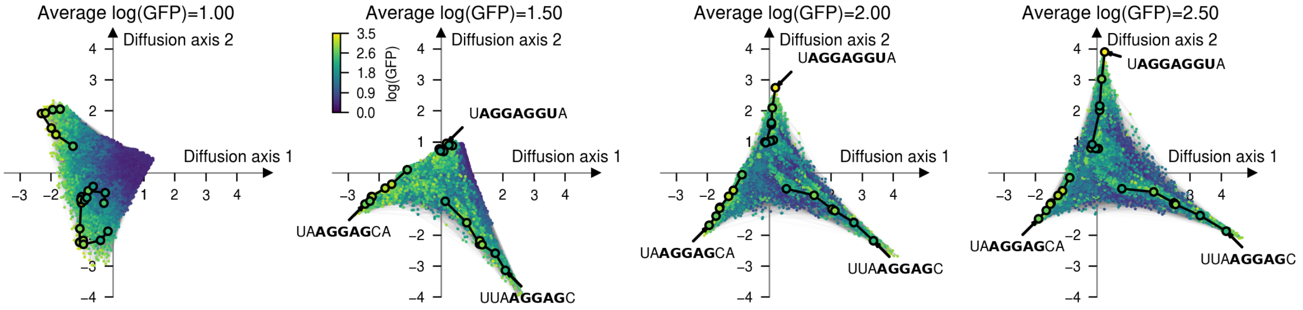

Fig. S7. Low-dimensional representation of the Shine-Dalgarno genotype-phenotype map inferred with VC regression from MAVE data along Diffusion axes 1 and 2 as a function of the assumed average log(GFP) at the stationary distribution (as determined by tuning the strength of selection parameter c). Every dot represents one of the 4^9 possible sequences and is colored according to its inferred log(GFP) values. Dots are plotted in order according to Diffusion axes 3.

bioRxiv preprint doi: https://doi.org/10.1101/2025.03.09.642267; this version posted August 26, 2025. The copyright holder for this preprint (which was not certified by peer review) is the author/funder, who has granted bioRxiv a license to display the preprint in perpetuity. It is made available under aCC-BY 4.0 International license.

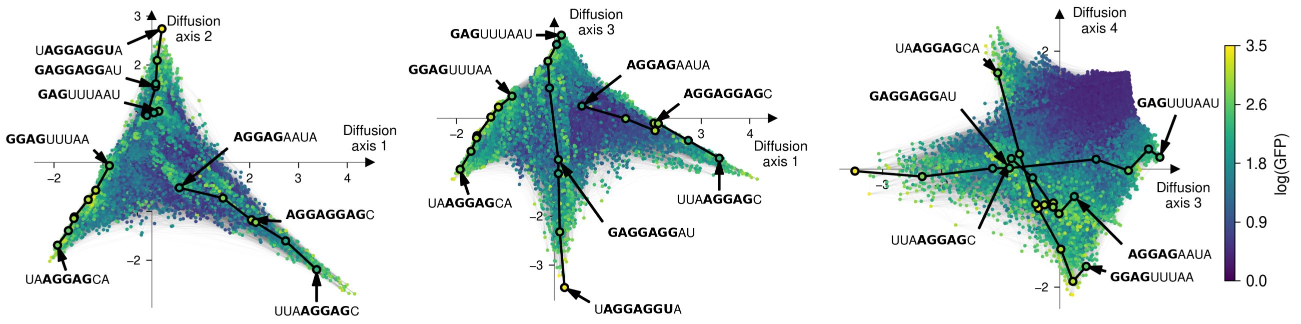

Fig. S8. Low-dimensional representation of the Shine-Dalgarno genotype-phenotype map inferred with VC regression from MAVE data along Diffusion axes 1, 2, 3 and 4. Every dot represents one of the possible 4^9 possible sequences and is colored according to its inferred log(GFP) value. Sequences are laid out according to the indicated Diffusion axes and dots are plotted in order according to Diffusion axes 3, 2 and 1, respectively for each panel.

bioRxiv preprint doi: https://doi.org/10.1101/2025.03.09.642267; this version posted August 26, 2025. The copyright holder for this preprint (which was not certified by peer review) is the author/funder, who has granted bioRxiv a license to display the preprint in perpetuity. It is made available under aCC-BY 4.0 International license.

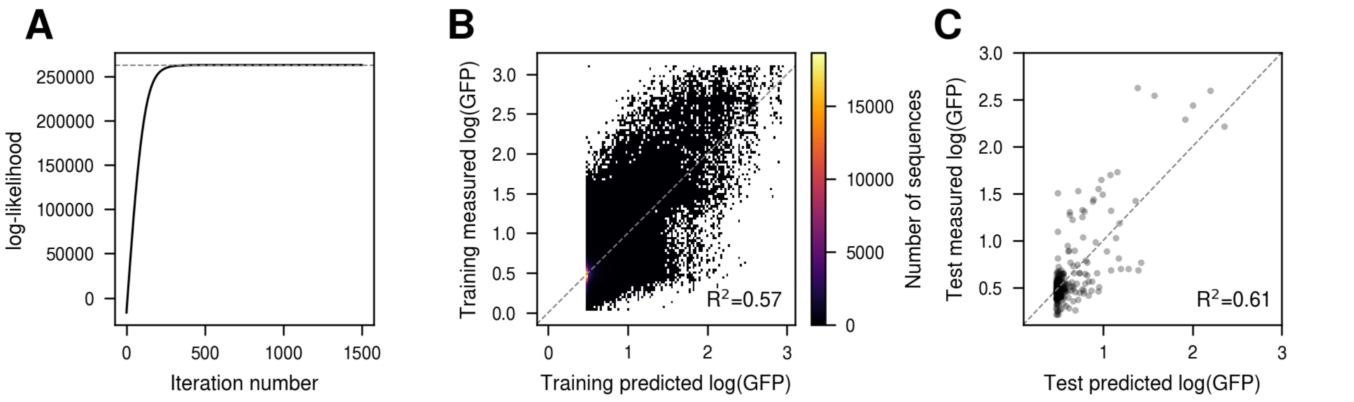

Fig. S9. Fitting a thermodynamic model to the Shine-Dalgarno genotype-phenotype map using MAVE data. (A) Training curve showing the evolution of the log-likelihood as a function of the number of iterations of the Adam optimizer. (B) Comparison of measured log(GFP) in the training data with the predicted values under the estimated thermodynamic model. (C) Comparison of measured log(GFP) in the test data with the predicted values under the estimated thermodynamic model.

bioRxiv preprint doi: https://doi.org/10.1101/2025.03.09.642267; this version posted August 26, 2025. The copyright holder for this preprint (which was not certified by peer review) is the author/funder, who has granted bioRxiv a license to display the preprint in perpetuity. It is made available under aCC-BY 4.0 International license.

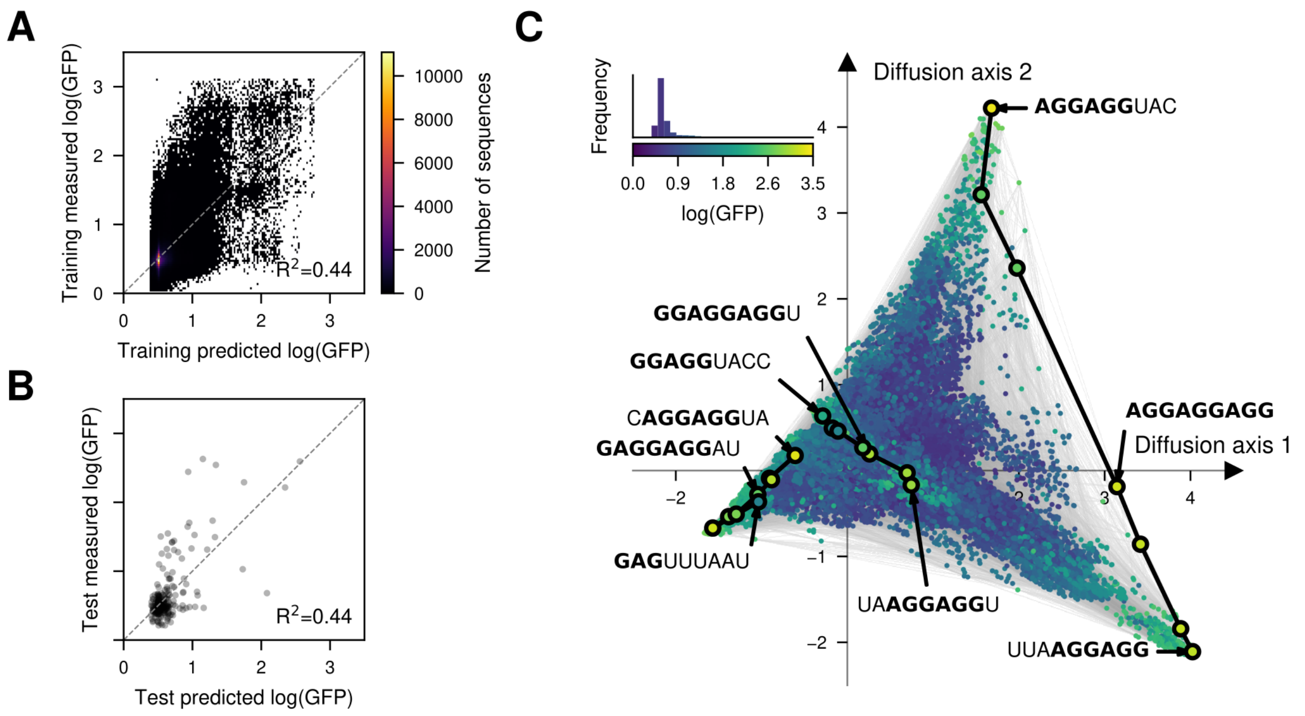

Fig. S10. A general thermodynamic model for RNA folding for predicting MAVE data for the Shine-Dalgarno sequence. (A,B) Comparison of measured log(GFP) in the training (A) and test data (B) with the predicted values under the calibration model using RNAcofold ensemble binding energies with the anti-SD sequence. (C) Visualization of the genotype-phenotype map that results from predicting the phenotype of every possible sequence RNAcofold ensemble binding energies to the anti-SD sequence. Every dot represents one of the possible 4^9 possible sequences and is colored according to the predicted log(GFP). The inset represents the phenotypic distribution along with their corresponding color in the map. Sequences are laid out according to the first two Diffusion axes and dots are plotted in order according to Diffusion axis 3.

bioRxiv preprint doi: https://doi.org/10.1101/2025.03.09.642267; this version posted August 26, 2025. The copyright holder for this preprint (which was not certified by peer review) is the author/funder, who has granted bioRxiv a license to display the preprint in perpetuity. It is made available under aCC-BY 4.0 International license.

Supplementary Information

A. Properties of the P_U projection operators. Let f represent a genotype-phenotype map on the space of sequences with a single site and α different alleles. The function f can be projected into a subspace spanned by any vector b using the orthogonal projection matrix $P = b(b^T b)^{-1} b$. Letting V_{con} be the constant subspace spanned by the α-dimensional vector of ones $b = \vec{1}$, the orthogonal projection matrix into V_{con} is thus given by $P_{con} = \frac{1}{\alpha}\vec{1}\vec{1}^T$. Let V_{add} be the orthogonal subspace ($V_{con} \perp V_{add}$), defined by its projection matrix $P_{add} = I - P_{con} = I - \frac{1}{\alpha}\vec{1}\vec{1}^T$. Thus, for any pair of sequences x, y, $P_{con}(x,y) = \frac{1}{\alpha}$ and

$$P_{add}(x,y) = \begin{cases} \frac{-1}{\alpha} & \text{if } x \neq y \\ \frac{\alpha-1}{\alpha} & \text{if } x = y. \end{cases}$$

For a genotype-phenotype map f in the space of sequences of length ℓ, these elementary subspaces can be combined through tensor products into 2^ℓ different $V_U = \bigotimes_p^\ell V_p$ subspaces defined by the set of sites U, such that $V_p = V_{add}$ for $p \in U$ and $V_p = V_{con}$ for $p \notin U$. Thus, the projection operator into the subspaces defined by U are obtained through the Kronecker product $P_U = \bigotimes_p^\ell P_p$, the elements of which are

$$P_U(x,y) = \alpha^{-\ell} \prod_{\substack{p\in U \\ x_p \neq y_p}} (-1) \prod_{\substack{p \in U \\ x_p = y_p}} (\alpha - 1). \tag{4}$$

It is easy to show that the resulting subspaces V_U are orthogonal to each other using the mixed product property of the Kronecker product $(A \otimes B)(C \otimes D) = (AC) \otimes (BD)$. As a consequence, if either $AC = 0$ or $BD = 0$, then $A \otimes B$ and $C \otimes D$ are orthogonal. If we consider two subspaces defined by different subsets of sites U and U', there will be at least one position p at which the two site-specific elementary subspaces are orthogonal to each other, i.e. $P_p P'_p = 0$. Consequently, $P_U P'_U = (\bigotimes_p^\ell P_p)(\bigotimes_p^\ell P'_p) = \bigotimes_p^\ell (P_p P'_p) = 0$.

Next, we consider the subspaces defined by the direct sum of subspaces V_U defined by exactly k sites $V_k = \bigoplus_{U:|U|=k} V_U$ and derive the corresponding projection operator P_k:

$$\begin{aligned} P_k(x,y) &= \sum_{U:|U|=k} P_U(x,y) = \sum_{U:|U|=k} \alpha^{-\ell} \prod_{\substack{p\in U \\ x_p \neq y_p}} (-1) \prod_{\substack{p \in U \\ x_p = y_p}} (\alpha - 1) \\ &= \alpha^{-\ell} \sum_{U:|U|=k} \prod_{\substack{p\in U \\ x_p \neq y_p}} (-1) \prod_{\substack{p \in U \\ x_p = y_p}} (\alpha - 1). \end{aligned} \tag{5}$$

We note that the elements in the sum are obtained by multiplying the factors $(\alpha - 1)$ and (-1) together a total of k times. These products can take only $k+1$ possible values: $(-1)^q(\alpha-1)^{\ell-q}$ for $q = 0, 1, \ldots, \ell$, where q represents the number of missmatches between sequences x and y within sets of sites U. Therefore, P_k can be expressed by:

$$P_k(x,y) = \alpha^{-\ell} \sum_q^k (-1)^q (\alpha-1)^{k-q} n_q(x,y), \tag{6}$$

where $n_q(x,y)$ is the number of times each unique value appears when summing through the corresponding P_U matrices. Because we are summing over all possible U of the same size, $n_q(x,y)$ does not depend on the specific sites or alleles at which x and y differ, but only on the Hamming distance between them $d(x,y)$. Specifically $n_q(x,y)$ is obtained by multiplying the number of ways to select q sites within the set of $d(x,y)$ different sites by the number of ways to select the remaining $k-q$ sites within the set of $\ell - d(x,y)$ matching sites:

$$P_k(x,y) = \alpha^{-\ell} \sum_q^k (-1)^q (\alpha-1)^{k-q} \binom{d(x,y)}{q} \binom{\ell - d(x,y)}{k-q} = \alpha^{-\ell} \mathcal{K}_k(d(x,y); \ell, \alpha), \tag{7}$$

where $\mathcal{K}_k(d(x,y);\ell,\alpha)$ denote the Krawtchouk polynomials (Stadler *et al.*, 1994; Zhou *et al.*, 2022). Note that this expression corresponds to the projection operator P_k into the subspace of pure k-th order interactions. Therefore, this shows that this k-th order interaction subspace V_k can be decomposed into smaller orthogonal subspaces V_U corresponding to pure k-th order interactions involving specific subsets of sites.

It is also easy to show that the columns of P_U are in the k-th eigenspace of the graph Laplacian by taking its projection and using the orthogonality properties of the P_U:

bioRxiv preprint doi: https://doi.org/10.1101/2025.03.09.642267; this version posted August 26, 2025. The copyright holder for this preprint (which was not certified by peer review) is the author/funder, who has granted bioRxiv a license to display the preprint in perpetuity. It is made available under aCC-BY 4.0 International license.

$$P_k P_U = \sum_{U':|U'|=k} P_{U'} P_U = \begin{cases} P_U & \text{if } |U| = k \\ 0 & \text{if } |U| \neq k. \end{cases} \tag{8}$$

Using the same argument, we can show that $P_U P_k = P_U \sum_{U':|U'|=k} P_{U'} = P_U$ when $|U| = k$ and 0 otherwise.

B. Variance explained by interactions among sets of sites. In section A, we introduced the projection matrix P_U. This enables decomposing a genotype-phenotype map f into its 2^ℓ components f_U, each representing pure $|U|$-order interactions among sites in a set U. Here, we present two coarse-grained views of these components by quantifying the percentage variance explained by genetic interactions involving specific sites in a genotype-phenotype map f, where the total variance is given by

$$\mathrm{Var}[f] = \alpha^{-\ell}(f - \bar{f})^T (f - \bar{f}), \tag{9}$$

where $\bar{f} = \alpha^{-\ell} \sum_i f_i$. In the first approach, we consider the variance explained by interactions of order k involving each site p in the sequence:

$$\mathrm{Var}_k^p[f] = \alpha^{-\ell}(f_k^p)^T f_k^p, \tag{10}$$

where

$$f_k^p = \sum_{\substack{U:p\in U \\ |U|=k}} P_U f. \tag{11}$$

The values $100 \times \frac{\mathrm{Var}_k^p[f]}{\mathrm{Var}[f]}$ for every combination of k and p can be arranged in an $\ell \times \ell$-dimensional matrix to reveal which sites contribute most to variance, and how much through additive, low-order or higher-order genetic interactions.

In the second approach, we consider the variance explained by interactions of order $k = 2$ and $k > 2$ involving pairs of sites p, q:

$$\mathrm{Var}_2^{p,q}[f] = \alpha^{-\ell}(f_2^{p,q})^T f_2^{p,q}, \tag{12}$$

$$\mathrm{Var}_{k>2}^{p,q}[f] = \alpha^{-\ell}(f_{k>2}^{p,q})^T f_{k>2}^{p,q}, \tag{13}$$

$$f_2^{p,q} = \sum_{\substack{U:\{p,q\}\subseteq U \\ |U|=2}} P_U f, \tag{14}$$

$$f_{k>2}^{p,q} = \sum_{\substack{U:\{p,q\}\subseteq U \\ |U|>2}} P_U f, \tag{15}$$

These values can be summarized in an $\ell \times \ell$-dimensional matrix M

$$M_{p,q}(f) = \begin{cases} 100 \times \frac{\mathrm{Var}_2^{p,q}[f]}{\mathrm{Var}_2[f]} & \text{if } p < q \\ 0 & \text{if } p = q \\ 100 \times \frac{\mathrm{Var}_{k>2}^{p,q}[f]}{\sum_{k>2} \mathrm{Var}_k[f]} & \text{if } p > q, \end{cases} \tag{16}$$

where $\mathrm{Var}_k[f] = f_k^T f_k$. This matrix reveals the interaction patterns between pairs of sites, distinguishing pairwise from higher-order effects.

C. Linear operator for Kronecker products. In section A, we describe the projection matrix P_U that projects a function f into the subspace corresponding to pure interactions between sites in a set U. Here we describe an efficient method for calculating the product $P_U f$ by noting that P_U can be written as a Kronecker product of ℓ matrices ($P_U = \bigotimes_p^\ell P_p$).

Let A be an arbitrary matrix obtained through an ℓ-Kronecker product, i.e. $A = \bigotimes_p^\ell A_p$ with entries given by $A(x,y) = \prod_p A_p(x_p, y_p)$ and $\mathbf{B} \in \mathbb{R}^{(\alpha\times\alpha\times\ldots\times\alpha)}$ be a tensor with ℓ dimensions such that $\mathbf{B}_{x_1,x_2,\ldots,x_\ell} = b_x$ for any vector $b \in \mathbb{R}^{\alpha^\ell}$. Thus,

bioRxiv preprint doi: https://doi.org/10.1101/2025.03.09.642267; this version posted August 26, 2025. The copyright holder for this preprint (which was not certified by peer review) is the author/funder, who has granted bioRxiv a license to display the preprint in perpetuity. It is made available under aCC-BY 4.0 International license.

any matrix vector product Ab can be computed without explicitly constructing A using tensor dot products as follows:

$$\begin{aligned}(Ab)_x &= \sum_y A(x,y)b_y \\ &= \sum_{y_1,\dots,y_\ell}\left(\prod_{p=1}^{\ell} A_p(x_p,y_p)\right)\mathbf{B}_{y_1,\dots,y_\ell} \\ &= \sum_{y_2,\dots,y_\ell}\left(\prod_{p=2}^{\ell} A_p(x_p,y_p)\right)\sum_{y_1} A_1(x_1,y_1)\mathbf{B}_{y_1,\dots,y_\ell}.\end{aligned} \tag{17}$$

Let $\mathbf{B}^{(1)}_{x_1,y_2,\dots,y_\ell} = \sum_{y_1} A_1(x_1,y_1)\mathbf{B}_{y_1,\dots,y_\ell}$ and repeat the same operation:

$$\begin{aligned}(Ab)_x &= \sum_{y_2,\dots,y_\ell}\left(\prod_{p=2}^{\ell} A_p(x_p,y_p)\right)\mathbf{B}^{(1)}_{x_1,\dots,y_\ell} \\ &= \sum_{y_3,\dots,y_\ell}\left(\prod_{p=3}^{\ell} A_p(x_p,y_p)\right)\sum_{y_2} A_2(x_2,y_2)\mathbf{B}^{(1)}_{x_1,y_2,\dots,y_\ell} \\ &= \sum_{y_3,\dots,y_\ell}\left(\prod_{p=3}^{\ell} A_p(x_p,y_p)\right)\mathbf{B}^{(2)}_{x_1,x_2,y_3,\dots,y_\ell}.\end{aligned} \tag{18}$$

Thus, it is easy to see that $(Ab)_x$ corresponds to $\mathbf{B}^{(\ell)}_{x_1,\dots,x_\ell}$ under the general recursion

$$\mathbf{B}^{(i)}_{x_1,\dots,x_i,y_{i+1},\dots,x_\ell} = \sum_{y_i} A_i(x_i,y_i)\mathbf{B}^{(i-1)}_{x_1,\dots,x_{i-1},y_i,\dots,y_\ell}, \tag{19}$$

where $\mathbf{B}^{(0)}_{y_1,\dots,y_\ell} = \mathbf{B}_{y_1,\dots,y_\ell} = b_y$. Note that each of the ℓ steps in the recursion can be computed efficiently as a tensor dot product between an $\alpha \times \alpha$ matrix and an $\alpha_1 \times_2 \alpha \times \cdots \times \alpha_\ell$ tensor requiring $\alpha^{\ell-1} \times \alpha^2$ operations. The total number of required operations, $\ell\alpha^{\ell+1}$, is much smaller than the $\alpha^{2\ell}$ operations required for naively computing Ab, but more importantly, this strategy reduces memory requirements from the prohibitive scaling with $\alpha^{2\ell}$ for storing A to only α^ℓ for storing $\mathbf{B}$, enabling practical computation for α^ℓ in the order of millions.

D. Linear operator for Laplacian of the Hamming graph. The space of possible sequences of length ℓ and α different alleles can be represented by a Hamming graph, in which nodes represent genotypes and edges represent single point mutations. The Laplacian matrix L of this graph is given by

$$L(i,j) = \begin{cases} -1 & \text{if } i \text{ and } j \text{ are neighbors,} \\ \ell(\alpha-1) & \text{if } i = j, \\ 0 & \text{otherwise.} \end{cases} \tag{20}$$

This matrix is sparse and can be stored in Compressed Sparse Row (CSR) format to efficiently compute matrix vector products. Despite the sparsity, there are still $\alpha^\ell \times (1+\ell(\alpha-1))$ non-zero entries. For a the space of sequences of length 9 with 4 alleles with 64 bits floating point values, only storing the non-zero entries would require about 450MB. Here, we develop a matrix-free function to compute matrix-vector products with the Laplacian matrix Lb by leveraging the highly regular structure of this matrix and tensor broadcasting, resulting in memory requirements that scale only with the size of sequence space α^ℓ. In particular, we can express Lb as

$$Lb = (\ell(\alpha-1)I - A)b = (\ell\alpha I - (\ell I + A))b = \ell\alpha b - (\ell I + A)b = \ell\alpha b - w,$$

where A is the adjacency matrix and w is the product $(\ell I + A)b$. For a fixed choice of b, let $\mathbf{B} \in \mathbb{R}^{(\alpha\times\alpha\times\dots\times\alpha)}$ be a tensor with ℓ dimensions such that $\mathbf{B}_{x_1,x_2,\dots,x_\ell} = b_x$, where x represents a sequence and x_i the allele at position i. Then for the same choice of b, the tensor $\mathbf{W}$ having elements $\mathbf{W}_{x_1,x_2,\dots,x_\ell} = w_x$ can be easily computed in tensor form using broadcasting by using the trick

$$\mathbf{W} = \sum_i^{\ell} \mathbf{B}^{(i)}. \tag{21}$$

bioRxiv preprint doi: https://doi.org/10.1101/2025.03.09.642267; this version posted August 26, 2025. The copyright holder for this preprint (which was not certified by peer review) is the author/funder, who has granted bioRxiv a license to display the preprint in perpetuity. It is made available under aCC-BY 4.0 International license.

$\mathbf{B}^{(i)}_{x_1,\dots,x_{i-1},*,x_{i+1},\dots,x_\ell} = \sum_c^\alpha \mathbf{B}_{x_1,\dots,x_{i-1},c,x_{i+1},\dots,x_\ell}$, where the '*' character indicates broadcasting, i.e. all characters at position i lead to the same value. Thus, this can be efficiently computed by summing the entries of tensor $\mathbf{B}$ over axis i. We can then use $\mathbf{B}$ and $\mathbf{W}$ to calculate $w = (\ell I + A)b$ as:

$$\begin{aligned}
w_x = \mathbf{W}_{x_1,x_2,\dots,x_\ell} &= \sum_i^\ell \sum_c^\alpha \mathbf{B}_{x_1,\dots,x_{i-1},c,x_{i+1},\dots,x_\ell} \\
&= \ell \mathbf{B}_{x_1,x_2,\dots,x_\ell} + \sum_i^\ell \sum_{c \neq x_i}^\alpha \mathbf{B}_{x_1,\dots,x_{i-1},c,x_{i+1},\dots,x_\ell} \\
&= \ell b_x + (Ab)_x = ((\ell I + A)b)_x.
\end{aligned}$$

E. Minimum epistasis interpolation solution and posterior distribution. In this section, we derive the minimum epistasis interpolation solution as the maximum a posteriori (MAP) estimate of a Gaussian process model under a prior distribution on local epistatic coefficients. Let us consider a complete genotype-phenotype map given by an α^ℓ-dimensional vector f and define an improper prior distribution defined by the precision matrix $C = \frac{a}{s}\Delta^P$, such that $\log \mathrm{p}(f) \propto -f^T C f$.

Assuming we know exactly the phenotypes f_x for a subset of sequences x, we aim to compute the posterior probability of the phenotypes f_z at all remaining unobserved sequences z given by $\mathrm{p}(f_z|f_x)$. Let us define the joint log-probability distribution over $[f_x, f_z]$ given by

$$\begin{aligned}
\log \mathrm{p}(f) = \log p\left(\begin{bmatrix} f_x \\ f_z \end{bmatrix}\right) \propto -\frac{1}{2} f^T C f = -\frac{1}{2}\begin{bmatrix} f_x^T & f_z^T \end{bmatrix} \begin{bmatrix} C_{xx} & C_{xz} \\ C_{xz} & C_{zz} \end{bmatrix} \begin{bmatrix} f_x \\ f_z \end{bmatrix} \\
= -\frac{1}{2}\left(f_x^T C_{xx} f_x + 2 f_x^T C_{xz} f_z + f_z^T C_{zz} f_z\right).
\end{aligned}$$

Since C_{zz} is a principal submatrix of the positive semidefinite matrix C, it is also positive semidefinite for any z, and thus $\log \mathrm{p}(f_z|f_x)$ is convex. We can now take the gradient $\nabla_{f_z} \log \mathrm{p}(f_z|f_x) = f_x^T C_{xz} f_z + C_{zz} f_z$ and find the MAP $\hat{f}_z$ as the solution to the equation $C_{zz}\hat{f}_z + C_{zx} f_x = 0$, which is unique if and only if the corresponding $P-1$-th order model is uniquely determined (Zhou and McCandlish, 2020) and is given by $\hat{f}_z = -C_{zz}^{-1} C_{zx} f_x$. In this case, it is easy to verify that the posterior covariance is given by C_{zz}^{-1} as

$$\begin{aligned}
\log \mathrm{p}(f_z|f_x) \propto (f_z + C_{zz}^{-1} C_{zx} f_x)^T C_{zz} (f_z + C_{zz}^{-1} C_{zx} f_x) = \\
= f_z^T C_{zz} f_z + 2 f_x^T C_{xz} C_{zz}^{-1} C_{zz} f_z + f_x^T C_{xz} C_{zz}^{-1} C_{zz} C_{zz}^{-1} C_{zx} f_x \\
= f_z^T C_{zz} f_z + 2 f_x^T C_{xz} f_z + f_x^T C_{xz} C_{zz}^{-1} C_{zx} f_x \propto f_z^T C_{zz} f_z + 2 f_x^T C_{xz} f_z.
\end{aligned} \tag{22}$$

In the case in which $C = \frac{a}{s}\Delta^{(P)}$, the posterior mean $\hat{f}_z = -(\Delta_{zz}^{(P)})^{-1}\Delta_{zx}^{(P)} f_x$ is independent of a. The posterior covariance, on the other hand, is independent of f_x, but depends on a and the pattern of observations. Thus, if we want to compute the variance of the phenotypic predictions of specific sequences, we define the a^* such that the expected sum of squared local epistatic coefficients $\mathbb{E}[\overline{\epsilon_P^2}] = \frac{1}{a}\mathrm{rank}(\Delta^{(P)})$ under the prior (Chen *et al.*, 2021) matches the one under the posterior mean given by $\frac{1}{s_P}\hat{f}^T \Delta^{(P)} \hat{f}$.

F. Posterior distribution computation with the cost matrix. Given a set of n measurements y in a subset of sequences x with measurement variances arranged along the diagonal of an $n \times n$ matrix D, we aim to obtain the complete genotype-phenotype map represented by the α^ℓ-dimensional vector $\hat{f}$ that maximizes the posterior probability of f given the observations y, i.e. we wish to find

$$\hat{f} = \arg\max_f \log \mathrm{p}(f|y).$$

We begin by defining an $\alpha^\ell \times n$ matrix X relating the points in the complete space with the n observed values, such that $f_x = X^T f$

$$X_{ij} = \begin{cases} 1 & \text{if observation } j \text{ corresponds to sequence } i \\ 0 & \text{otherwise.} \end{cases}$$

Using X, we can then write the posterior log-probability as a function of f:

bioRxiv preprint doi: https://doi.org/10.1101/2025.03.09.642267; this version posted August 26, 2025. The copyright holder for this preprint (which was not certified by peer review) is the author/funder, who has granted bioRxiv a license to display the preprint in perpetuity. It is made available under aCC-BY 4.0 International license.

$$\log \mathrm{p}(f|y) \propto -\frac{1}{2} f^T C f - \frac{1}{2}(y - X^T f)^T D^{-1}(y - X^T f). \tag{23}$$

We can then expand this expression to separate factors that depend on f from those that depend only on the data y.

$$\begin{aligned}\log \mathrm{p}(f|y) &\propto -\frac{1}{2} f^T C f - \frac{1}{2} y^T D^{-1} y + f^T X D^{-1} y - \frac{1}{2} f^T X D^{-1} X^T f \\ &= -\frac{1}{2} f^T (C + X D^{-1} X^T) f + f^T X D^{-1} y - \frac{1}{2} y^T D^{-1} y.\end{aligned}$$

We next take the gradient with respect to f

$$\nabla_f \log \mathrm{p}(f|y) = -(C + X D^{-1} X^T) f + X D^{-1} y,$$

and solve for $\hat{f}$ by setting $\nabla_f \log \mathrm{p}(f|y) = 0$, which yields

$$\hat{f} = (C + X D^{-1} X^T)^{-1} X D^{-1} y. \tag{24}$$

If C is invertible, then we can define a kernel matrix $K = C^{-1}$ over the complete genotype-phenotype map and verify that this is equivalent to the classical solution for the posterior mean of a Gaussian process model using Woodbury's identity. Specifically, for a subset of sequences z and the α^ℓ by $|z|$ matrix Z defined by:

$$Z_{ij} = \begin{cases} 1 & \text{if sequence } i \text{ is the } j\text{-th member of } z \\ 0 & \text{otherwise,} \end{cases}$$

we find that

$$\begin{aligned}\hat{f}_z &= Z^T \hat{f} \\ &= Z^T (C + X D^{-1} X^T)^{-1} X D^{-1} y \\ &= Z^T \left(K - K X (X^T K X + D)^{-1} X^T K\right) X D^{-1} y \\ &= Z^T K \left(I - X (X^T K X + D)^{-1} X^T K\right) X D^{-1} y \\ &= Z^T K \left(X - X (X^T K X + D)^{-1} X^T K X\right) D^{-1} y \\ &= Z^T K X \left(I - (X^T K X + D)^{-1} X^T K X\right) D^{-1} y.\end{aligned}$$

Then we can use the fact that $I = (X^T K X + D)^{-1}(X^T K X + D)$ to obtain the identity:

$$\begin{aligned}&I - (X^T K X + D)^{-1} X^T K X \\ &= (X^T K X + D)^{-1}(X^T K X + D) - (X^T K X + D)^{-1} X^T K X \\ &= (X^T K X + D)^{-1}(X^T K X + D - X^T K X) \\ &= (X^T K X + D)^{-1} D.\end{aligned}$$

Substituting this identity into our previous expression for $\hat{f}_z$, we now recover the classical maximum a posteriori solution for Gaussian process regression

$$\hat{f}_z = Z^T K X \left(X^T K X + D\right)^{-1} D D^{-1} y = Z^T K X \left(X^T K X + D\right)^{-1} y,$$

as desired.

Turning to the covariance matrix for the posterior, knowing that the posterior distribution is multivariate Gaussian implies that the posterior covariance matrix is given by the inverse of the Hessian matrix of the log-posterior probability. Thus the covariance matrix of the posterior distribution is given by:

$$\Sigma = \left(\nabla\nabla_f log(f|y)\right)^{-1} = \left(C + X D^{-1} X^T\right)^{-1}, \tag{25}$$

bioRxiv preprint doi: https://doi.org/10.1101/2025.03.09.642267; this version posted August 26, 2025. The copyright holder for this preprint (which was not certified by peer review) is the author/funder, who has granted bioRxiv a license to display the preprint in perpetuity. It is made available under aCC-BY 4.0 International license.

which we note depends on our observations only through the pattern of observed sequence as encoded in X and not on the observed phenotypes y.

Based on the marginalization property of multivariate Gaussian distributions, the posterior covariance at a subset of points z can be obtained simply by taking the submatrix $\Sigma_{zz} = Z^T \Sigma Z$. We can verify that this also matches the classical solution for Gaussian process posterior covariance when $K = C^{-1}$ using Woodbury's identity:

$$\begin{aligned}\Sigma_{zz} &= Z^T \left(C + XD^{-1}X^T\right)^{-1} Z \\ &= Z^T \left(C^{-1} - C^{-1}\left(X^T C^{-1} X + D\right)^{-1} C^{-1}\right) Z \\ &= Z^T \left(K - K\left(X^T K X + D\right)^{-1} K\right) Z \\ &= Z^T K Z - Z^T K \left(X^T K X + D\right)^{-1} K Z \\ &= K_{zz} - K_{zx}\left(K_{xx} + D\right)^{-1} K_{xz},\end{aligned} \tag{26}$$

as desired.
