# Supplementary material for: Inference and visualization of complex genotype-phenotype maps with gpmap-tools
Source: bioRxiv. 2025 Mar 15:2025.03.09.642267. Preprint. [Version 2] doi: 10.1101/2025.03.09.642267 (PMC11952336; doi:10.1101/2025.03.09.642267)
Supplement: Supplementary file 1 [file NIHPP2025.03.09.642267v2-supplement-1.pdf]

bioRxiv preprint doi: https://doi.org/10.1101/2025.03.09.642267; this version posted March 15, 2025. The copyright holder for this preprint (which was not certified by peer review) is the author/funder, who has granted bioRxiv a license to display the preprint in perpetuity. It is made available under aCC-BY 4.0 International license.

Supplementary Figures

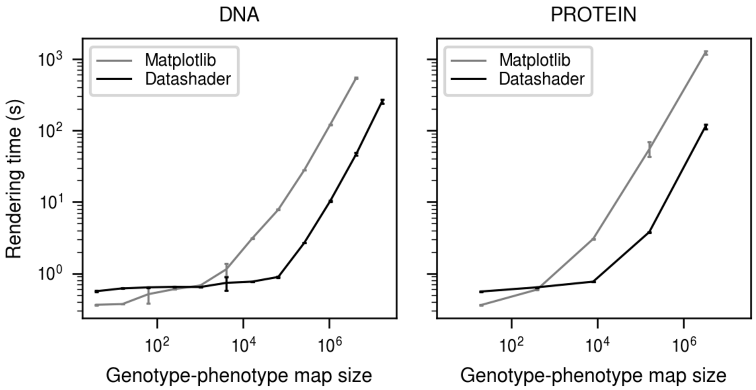

Fig. S1. Visualization rendering times using two different back-end libraries for plotting as a function of the size of DNA and protein genotype-phenotype maps.

bioRxiv preprint doi: https://doi.org/10.1101/2025.03.09.642267; this version posted March 15, 2025. The copyright holder for this preprint (which was not certified by peer review) is the author/funder, who has granted bioRxiv a license to display the preprint in perpetuity. It is made available under aCC-BY 4.0 International license.

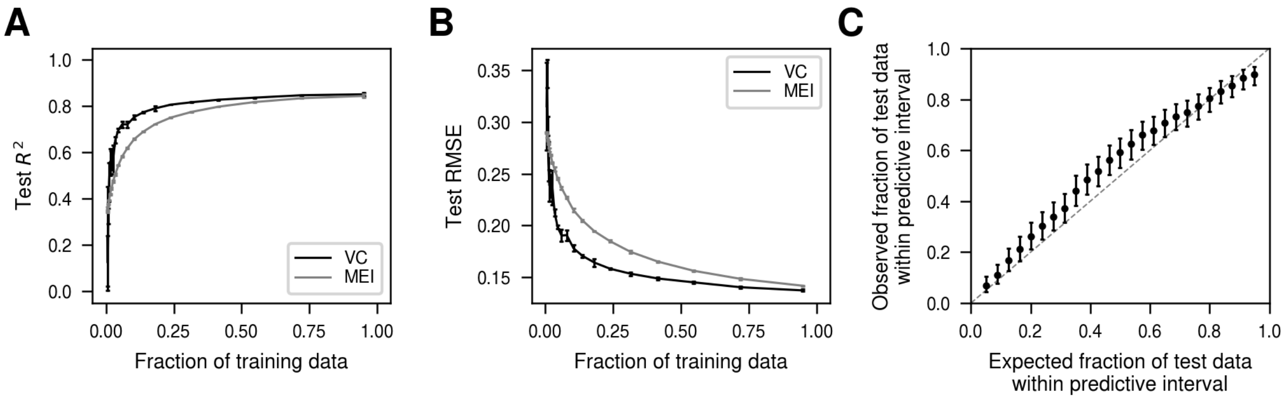

Fig. S2. Predictive performance of Minimum Epistasis Interpolation and Variance Component regression in held-out data. (A,B) Model predictive performance measured by the R^2 (A) and RMSE (B) in held-out data as a function of the fraction of data used for training and phenotypic prediction. Error bars represent the standard deviation over 3 independent subsets of sequences used for training at each proportion. (C) Evaluation of Variance Component regression model calibration by comparing the expected fraction of times a predictive interval will contain the real phenotypic value compared to the fraction of times it actually contained the measured phenotype across 274 test data points. Error bars represent the 95% Jeffreys confidence interval for the estimated fraction of data points laying within the corresponding predictive interval. Diagonal dashed gray line shows the expectation under perfect model calibration.

bioRxiv preprint doi: https://doi.org/10.1101/2025.03.09.642267; this version posted March 15, 2025. The copyright holder for this preprint (which was not certified by peer review) is the author/funder, who has granted bioRxiv a license to display the preprint in perpetuity. It is made available under aCC-BY 4.0 International license.

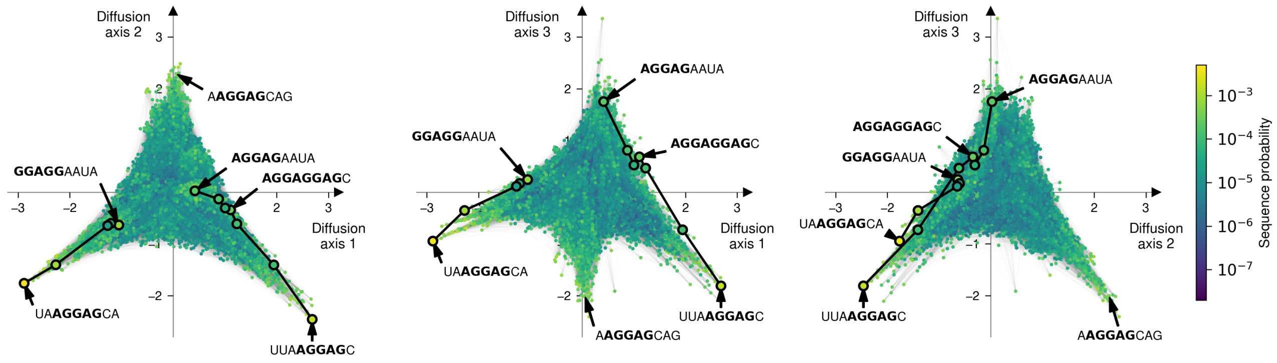

Fig. S3. Low dimensional representation of the Shine-Dalgarno probability distribution inferred with SeqDEFT along Diffusion axes 1, 2 and 3. Every dot represents one of the possible 4^9 possible sequences and is colored according to their inferred probability. Sequences are laid out according to the indicated Diffusion axes and dots are plotted in order according to the missing Diffusion axis.

bioRxiv preprint doi: https://doi.org/10.1101/2025.03.09.642267; this version posted March 15, 2025. The copyright holder for this preprint (which was not certified by peer review) is the author/funder, who has granted bioRxiv a license to display the preprint in perpetuity. It is made available under aCC-BY 4.0 International license.

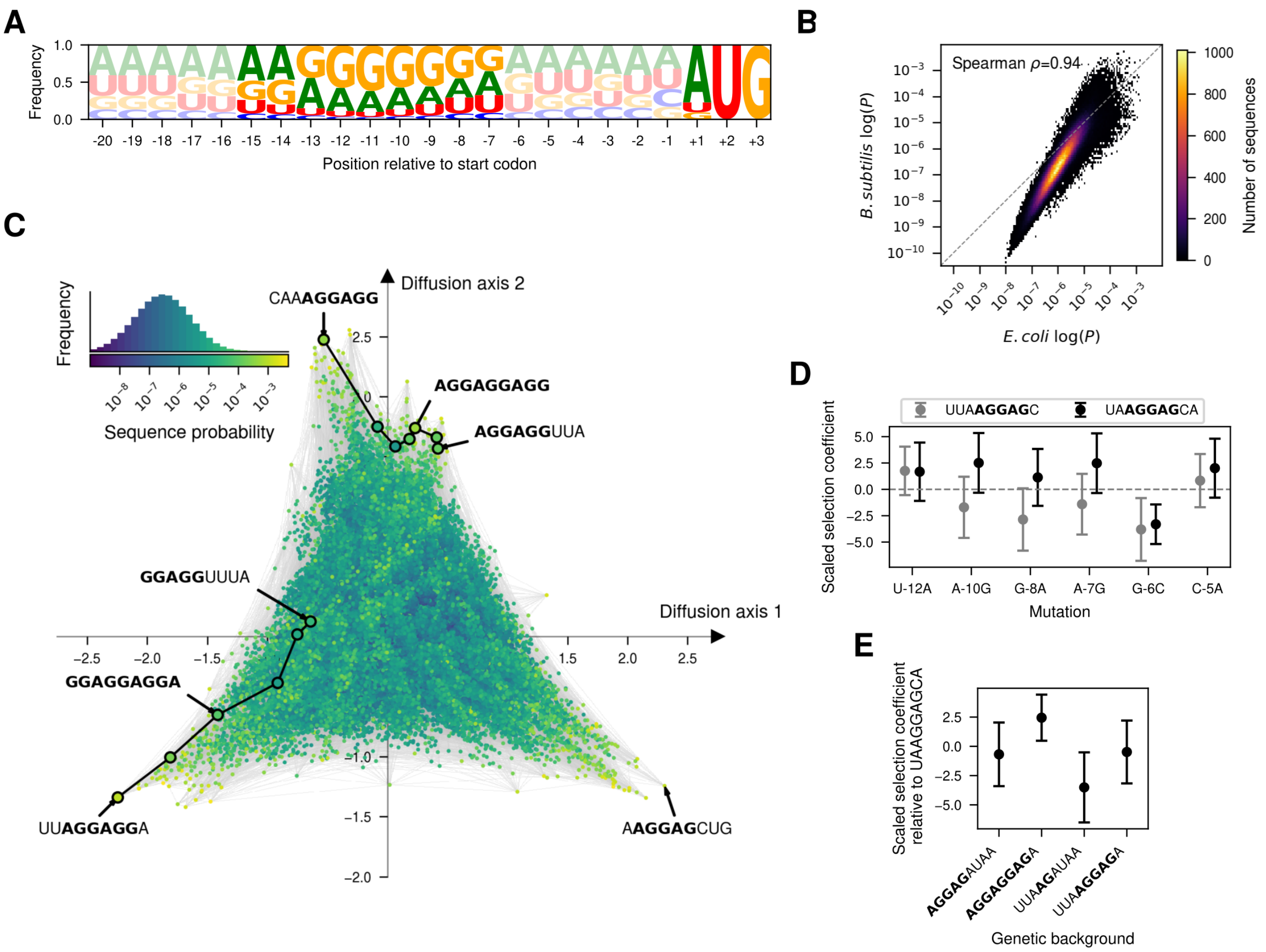

Fig. S4. The structure of the genotype-phenotype map inferred from *B. subtilis* is conserved. (A) Sequence logo representing the site-specific allele frequencies of 4,328 5'UTRs in the *B. subtilis* genome aligned with respect to the annotated start codon. The start codon and the 9 nucleotide sequences 4 bases upstream are highlighted to emphasize the most relevant cis-regulatory sequences for translation initiation. (B) Two-dimensional histogram representing the relationship between the inferred sequence probabilities from their frequency in the *E. coli* and *B. subtilis* genomes. (C) Low dimensional representation of the Shine-Dalgarno probability distribution inferred with SeqDEFT. Every dot represents one of the possible 4^9 possible sequences and is colored according to its inferred probability. The inset represents the distribution of inferred sequence probabilities along with their corresponding color in the visualization. Sequences are laid out according to the first two Diffusion axes and dots are plotted in order according to the 3rd Diffusion axis. (D) Posterior distribution of the effects of specific mutations when introduced in two genetic contexts UUAAGGAGC and UAAGGAGCA representing a shift by one position of the AGGAG motif. (E) Posterior distribution of phenotypes associated to genotypes representing the shift of the AGGAG motif by 3 positions. Points represent the Maximum a posteriori (MAP) estimates and error bars represent the 95% credible intervals.

bioRxiv preprint doi: https://doi.org/10.1101/2025.03.09.642267; this version posted March 15, 2025. The copyright holder for this preprint (which was not certified by peer review) is the author/funder, who has granted bioRxiv a license to display the preprint in perpetuity. It is made available under aCC-BY 4.0 International license.

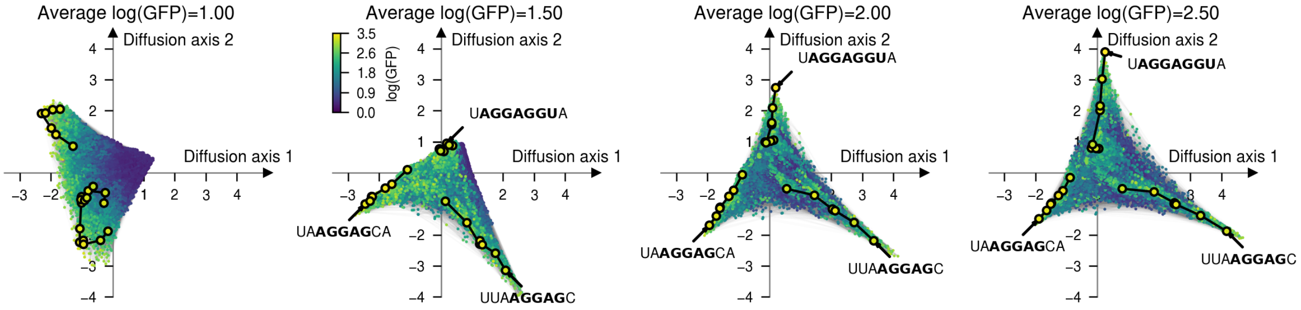

Fig. S5. Low-dimensional representation of the Shine-Dalgarno genotype-phenotype map inferred with VC regression from MAVE data along Diffusion axes 1 and 2 as a function of the assumed average log(GFP) at the stationary distribution (as determined by tuning the strength of selection parameter c). Every dot represents one of the 4^9 possible sequences and is colored according to its inferred log(GFP) values. Dots are plotted in order according to Diffusion axes 3.

bioRxiv preprint doi: https://doi.org/10.1101/2025.03.09.642267; this version posted March 15, 2025. The copyright holder for this preprint (which was not certified by peer review) is the author/funder, who has granted bioRxiv a license to display the preprint in perpetuity. It is made available under aCC-BY 4.0 International license.

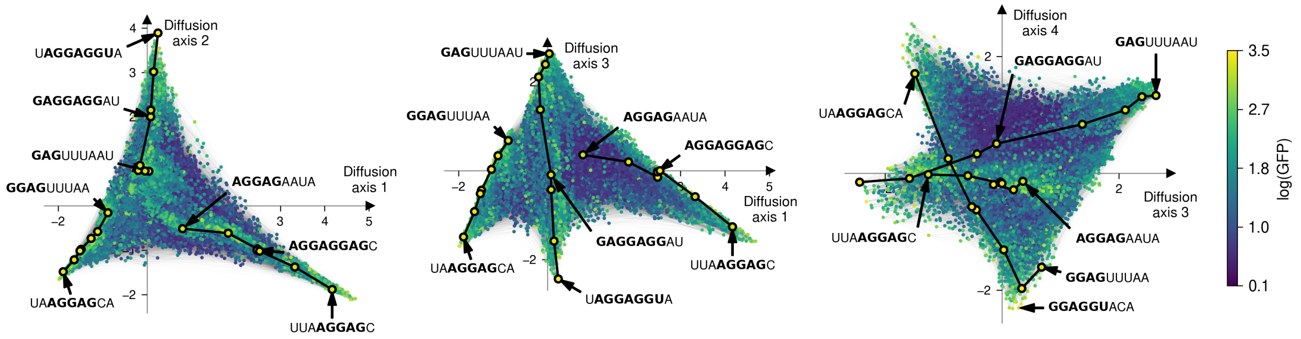

Fig. S6. Low dimensional representation of the Shine-Dalgarno genotype-phenotype map inferred with VC regression from MAVE data along Diffusion axes 1, 2, 3 and 4. Every dot represents one of the possible 4^9 possible sequences and is colored according to its inferred log(GFP) value. Sequences are laid out according to the indicated Diffusion axes and dots are plotted in order according to Diffusion axes 3, 2 and 1, respectively for each panel.

bioRxiv preprint doi: https://doi.org/10.1101/2025.03.09.642267; this version posted March 15, 2025. The copyright holder for this preprint (which was not certified by peer review) is the author/funder, who has granted bioRxiv a license to display the preprint in perpetuity. It is made available under aCC-BY 4.0 International license.

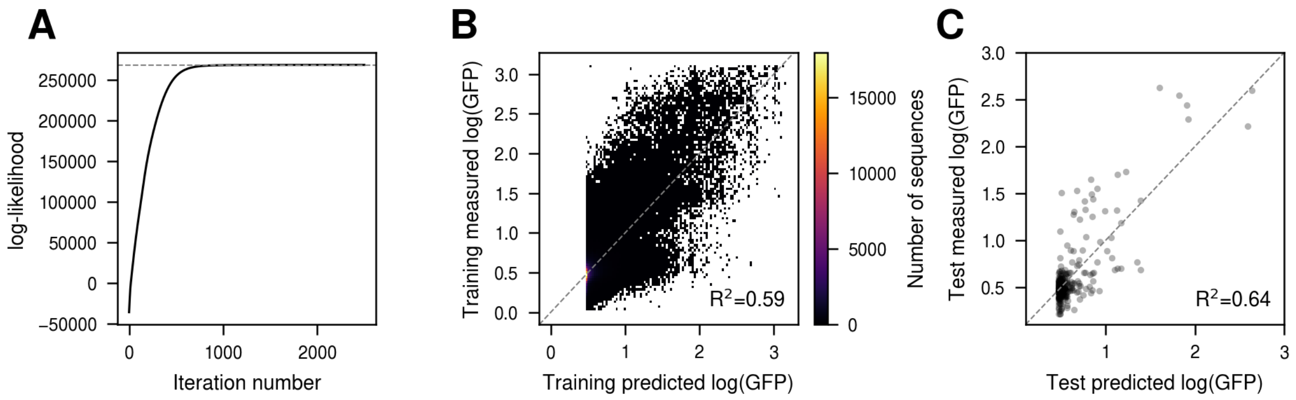

Fig. S7. Fitting a thermodynamic model to the Shine-Dalgarno genotype-phenotype map using MAVE data. (A) Training curve showing the evolution of the log-likelihood as a function of the number of iterations of the Adam optimizer. (B) Comparison of measured log(GFP) in the training data with the predicted values under the estimated thermodynamic model. (C) Comparison of measured log(GFP) in the test data with the predicted values under the estimated thermodynamic model.

bioRxiv preprint doi: https://doi.org/10.1101/2025.03.09.642267; this version posted March 15, 2025. The copyright holder for this preprint (which was not certified by peer review) is the author/funder, who has granted bioRxiv a license to display the preprint in perpetuity. It is made available under aCC-BY 4.0 International license.

Supplementary Information

Linear operator for Laplacian of the Hamming graph. The space of possible sequences of length ℓ and α different alleles can be represented by a Hamming graph, in which nodes represent genotypes and edges represent single point mutations. The Laplacian matrix L of this graph is given by

$$L(i,j) = \begin{cases} -1 & \text{if } i \text{ and } j \text{ are neighbors} \\ \ell(\alpha-1) & \text{if } i = j \\ 0 & \text{Otherwise} \end{cases} \quad \textbf{(14)}$$

This matrix is sparse and can be stored in Compressed Sparse Row (CSR) format to efficiently compute matrix vector products. Despite the sparsity, there are still $\alpha^\ell \times (1+\ell(\alpha-1))$ non-zero entries. For a the space of sequences of length 9 with 4 alleles with 64 bits floating point values, only storing the non-zero entries would require about 450MB. Here, we develop a matrix-free function to compute matrix-vector products with the Laplacian matrix Lb by leveraging the highly regular structure of this matrix and tensor broadcasting with memory requirements that scale only with the size of sequence space α^ℓ. We can express Lb as

$$Lb = (\ell(\alpha-1)I - A)b = (\ell\alpha I - (\ell I + A))b = \ell\alpha b - (\ell I + A)b = \ell\alpha b - w$$

where A is the adjacency matrix and w is the product $(\ell I + A)b$. For a fixed choice of b, let $\mathbf{B} \in \mathbb{R}^{(\alpha\times\alpha\times\ldots\times\alpha)}$ a tensor with ℓ dimensions such that $\mathbf{B}_{x_1,x_2,\ldots,x_\ell} = b_x$, where x represents a sequence and x_i the allele at position i. Then for the same choice of b, the tensor $\mathbf{W}$, where $\mathbf{W}_{x_1,x_2,\ldots,x_\ell} = w_x$, can be easily computed in tensor form using broadcasting,

$$\mathbf{W} = \sum_i^\ell \mathbf{B}^{(i)} \quad \textbf{(15)}$$

where $\mathbf{B}^{(i)}_{x_1,\ldots,x_{i-1},*,x_{i+1},\ldots,x_\ell} = \sum_c^\alpha \mathbf{B}_{x_1,\ldots,x_{i-1},c,x_{i+1},\ldots,x_\ell}$, which can be efficiently computed by summing the entries of $\mathbf{B}$ over axis i. We can then use $\mathbf{B}$ and $\mathbf{W}$ to calculate $w = (\ell I + A)b$ as:

$$\begin{aligned} w_x = \mathbf{W}_{x_1,x_2,\ldots,x_\ell} &= \sum_i^\ell \sum_c^\alpha \mathbf{B}_{x_1,\ldots,x_{i-1},c,x_{i+1},\ldots,x_\ell} \\ &= \ell\mathbf{B}_{x_1,x_2,\ldots,x_\ell} + \sum_i^\ell \sum_{c\neq x_i}^\alpha \mathbf{B}_{x_1,\ldots,x_{i-1},c,x_{i+1},\ldots,x_\ell} \\ &= \ell b_x + (Ab)_x = ((\ell I + A)b)_x. \end{aligned}$$

Properties of the P_U projection operators. Let f represent a genotype-phenotype map in the space of sequences with a single site and α different alleles. The function f can be projected into the constant subspace V_0 through the projection matrix $P_0 = b(b^Tb)^{-1}b = \frac{1}{\alpha}\vec{1}\vec{1}^T$ and into the orthogonal subspace through the projection matrix $P_1 = I - P_0 = I - \frac{1}{\alpha}\vec{1}\vec{1}^T$. For any pair of sequences x,y, $P_0(x,y) = \frac{1}{\alpha}$ and

$$P_1(x,y) = \begin{cases} \frac{-1}{\alpha} & \text{if } x \neq y \\ \frac{\alpha-1}{\alpha} & \text{if } x = y. \end{cases}$$

For a genotype-phenotype map f in the space of sequences of length ℓ, these elementary subspaces can be combined through tensor products into 2^ℓ different $V_U = \bigotimes_p^\ell V_p$ subspaces defined by the set of sites U, such that $V_p = V_1$ for $p \in U$ and $V_p = V_0$ for $p \notin U$. Thus, the projection operator into the subspaces defined by U are obtained through Kronecker product $P_U = \bigotimes_p^\ell P_p$

$$P_U(x,y) = \alpha^{-\ell} \prod_{\substack{p\in U \\ x_p\neq y_p}} (-1) \prod_{\substack{p\in U_p \\ x_p=y_p}} (\alpha-1). \quad \textbf{(16)}$$

It is easy to show that the resulting subspaces V_U are orthogonal to each other using the mixed product property of the Kronecker product $(A\otimes B)(C\otimes D) = (AC)\otimes(BD)$. As a consequence, if either $AC = 0$ or $BD = 0$, then $(A\otimes B)(C\otimes D) = 0$ are orthogonal. If we consider two subspaces defined by different subsets of sites U and U', there will be at least one position at which the two site-specific elementary subspaces are orthogonal to each other $P_pP_p' = P_0P_1 = 0$, such that $P_UP_U' = (\bigotimes_p^\ell P_p)(\bigotimes_p^\ell P_p') = \bigotimes_p^\ell (P_pP_p') = 0$. Next, we consider the subspaces defined by the direct sum of subspaces U containing exactly k sites $V_k = \bigoplus_{U:|U|=k} V_U$ and derive the corresponding projection operator P_k:

bioRxiv preprint doi: https://doi.org/10.1101/2025.03.09.642267; this version posted March 15, 2025. The copyright holder for this preprint (which was not certified by peer review) is the author/funder, who has granted bioRxiv a license to display the preprint in perpetuity. It is made available under aCC-BY 4.0 International license.

$$P_k(x,y) = \sum_{U:|U|=k} P_U(x,y) = \sum_{U:|U|=k} \alpha^{-\ell} \prod_{\substack{p\in U \\ x_p\neq y_p}} (-1) \prod_{\substack{p\in U_p \\ x_p=y_p}} (\alpha-1)$$
$$= \alpha^{-\ell} \sum_{U:|U|=k} \prod_{\substack{p\in U \\ x_p\neq y_p}} (-1) \prod_{\substack{p\in U_p \\ x_p=y_p}} (\alpha-1) \qquad \textbf{(17)}$$

We note that the elements in the sum can only be obtained by multiplying the factors $(\alpha-1)$ and (-1) k times. Therefore, these products can take only $k+1$ possible values $(\alpha-1)^0(-1)^l, (\alpha-1)^1(-1)^{\ell-1}, ..., (\alpha-1)^k(-1)^0$ or more generally $(-1)^q(\alpha-1)^{\ell-q}$, such that P_k can be expressed by:

$$P_k(x,y) = \alpha^{-\ell}\sum_q^k (-1)^q(\alpha-1)^{k-q} n_q, \qquad \textbf{(18)}$$

where n_q is the number of times each unique value appears when summing through the corresponding P_U matrices. Whether we take $(\alpha-1)$ or (-1) depends on whether sequences x and y have the same alleles at the sites in U, but not on which alleles. Moreover, because we are summing over all possible U of the same size, n_q does not depends on the specific sites that are different, but only on the Hamming distance $d(x,y)$ between sequences x and y. Specifically n_q can be obtained by multiplying the number of ways in which we can select q sites within the set of different sites $d(x,y)$, given by $\binom{d(x,y)}{q}$, with the number of ways we can select the $k-q$ sites within the set of $\ell-d(x,y)$ sites with the same allele, given by $\binom{\ell-d(x,y)}{k-q}$.

$$P_k(x,y) = \alpha^{-\ell}\sum_q^k (-1)^q(\alpha-1)^{k-q}\binom{d(x,y)}{q}\binom{\ell-d(x,y)}{k-q}. \qquad \textbf{(19)}$$

Note that this expression corresponds to the projection operator into the k-th order subspace (61), showing that the k-th order subspace can be decomposed into smaller subspaces V_U corresponding to pure k-th order interactions involving specific subsets of sites. It is also easy to show that the columns of P_U are in the k-th eigenspace of the graph Laplacian by taking its projection and using the P_U orthogonality properties:

$$P_kP_U = \sum_{U':|U'|=k} P_{U'}P_U = \begin{cases} P_U & \text{if } |U|=k \\ 0 & \text{if } |U|\neq k. \end{cases} \qquad \textbf{(20)}$$

Using the same argument, we can show that the columns of P_U span a non-zero subspace within the k-th order eigenspace of the graph Laplacian since $P_UP_k = P_U\sum_{U':|U'|=k}P_{U'}$ when $|U|=k$.

Linear operator for ℓ-Kronecker products. In the previous section, we describe the projection matrix P_U that projects a function f into the subspace corresponding to pure interactions between sites in a set U. Here we describe an efficient method for calculating the product P_Uf by noting that P_U can be written as a Kronecker product of ℓ matrices ($P_U = \bigotimes_p^\ell P_p$). Specifically, for any matrix A for any matrix obtained through an ℓ-Kronecker product $A = \bigotimes_p^\ell A_p$, we can compute the matrix vector product Ab without explicitly constructing A as follows:

$$Ab = (\bigotimes_{p=1}^{\ell} A_p)b = (A_1 \bigotimes_{p=2}^{\ell} A_p)b$$
$$= \begin{bmatrix} (A_1)_{11}(\bigotimes_{p=2}^\ell A_p)b_1 + (A_1)_{12}(\bigotimes_{p=2}^\ell A_p)b_2 + \cdots + (A_1)_{1\alpha}(\bigotimes_{p=2}^\ell A_p)b_\ell \\ (A_1)_{21}(\bigotimes_{p=2}^\ell A_p)b_1 + (A_1)_{22}(\bigotimes_{p=2}^\ell A_p)b_2 + \cdots + (A_1)_{2\alpha}(\bigotimes_{p=2}^\ell A_p)b_\ell \\ \vdots \\ (A_1)_{\alpha 1}(\bigotimes_{p=2}^\ell A_p)b_1 + (A_1)_{\alpha 2}(\bigotimes_{p=2}^\ell A_p)b_2 + \cdots + (A_1)_{\alpha\alpha}(\bigotimes_{p=2}^\ell A_p)b_\ell \end{bmatrix}$$
$$= \begin{bmatrix} \sum_c^\alpha (A_1)_{1c}(\bigotimes_{p=2}^\ell A_p)b_c \\ \sum_c^\alpha (A_1)_{2c}(\bigotimes_{p=2}^\ell A_p)b_c \\ \vdots \\ \sum_c^\alpha (A_1)_{\alpha c}(\bigotimes_{p=2}^\ell A_p)b_c \end{bmatrix} = \begin{bmatrix} (\bigotimes_{p=2}^\ell A_p)\sum_c^\alpha (A_1)_{1c}b_c \\ (\bigotimes_{p=2}^\ell A_p)\sum_c^\alpha (A_1)_{2c}b_c \\ \vdots \\ (\bigotimes_{p=2}^\ell A_p)\sum_c^\alpha (A_1)_{\alpha c}b_c \end{bmatrix} = \begin{bmatrix} (\bigotimes_{p=2}^\ell A_p)u_{11} \\ (\bigotimes_{p=2}^\ell A_p)u_{12} \\ \vdots \\ (\bigotimes_{p=2}^\ell A_p)u_{1\alpha} \end{bmatrix},$$

bioRxiv preprint doi: https://doi.org/10.1101/2025.03.09.642267; this version posted March 15, 2025. The copyright holder for this preprint (which was not certified by peer review) is the author/funder, who has granted bioRxiv a license to display the preprint in perpetuity. It is made available under aCC-BY 4.0 International license.

where we let $u_{1i} = \sum_c^\alpha (A_1)_{ic} b_c$ and note that these vectors can be computed simultaneously by multiplying an $\alpha \times \alpha$ matrix with an $\alpha \times \alpha^{\ell-1}$ matrix

$$\begin{bmatrix} u_{11}^T \\ u_{12}^T \\ \vdots \\ u_{1\alpha}^T \end{bmatrix} = \begin{bmatrix} \sum_c^\alpha (A_1)_{1c} b_c^T \\ \sum_c^\alpha (A_1)_{2c} b_c^T \\ \vdots \\ \sum_c^\alpha (A_1)_{\alpha c} b_c^T \end{bmatrix} = A_1 \begin{bmatrix} b_1^T \\ b_2^T \\ \vdots \\ b_\alpha^T \end{bmatrix}.$$

Once these vectors are computed, we can use the same strategy to compute the $(\bigotimes_{p=2}^{\ell} A_p) u_{1j}$. Recursively repeating this calculation, we find that we can compute Ab using only ℓ different $\alpha \times \alpha$ by $\alpha \times \alpha^{\ell-1}$ matrix multiplication operations, avoiding storage and computation of the $\alpha^\ell \times \alpha^\ell$ dense matrix A.

Posterior distribution computation with the cost matrix. Given a set of n measurements y in a subset of sequences x with measurement variances arranged along the diagonal of am $n \times n$ matrix D, we aim to obtain the complete genotype-phenotype map represented by the α^ℓ-dimensional vector $\hat{f}$ that maximizes the posterior probability of f given the observations y, i.e. we wish to find

$$\hat{f} = \arg\max_f \log p(f|y).$$

We begin by defining an $\alpha^\ell \times n$ matrix X relating the points in the complete space with the n observed values, such that $f_x = X^T f$

$$X_{ij} = \begin{cases} 1 & \text{if observation } j \text{ corresponds to sequence } i \\ 0 & \text{otherwise.} \end{cases}$$

Using X, we can then write the posterior log-probability as a function of f:

$$\log p(f|y) \propto -\frac{1}{2} f^T C f - \frac{1}{2} (y - X^T f)^T D^{-1} (y - X^T f). \tag{21}$$

We can then expand this expression to separate factors that depend on f from those that depend only on the data y.

$$\begin{aligned} \log p(f|y) &\propto -\frac{1}{2} f^T C f - \frac{1}{2} y^T D^{-1} y + f^T X D^{-1} y - \frac{1}{2} f^T X D^{-1} X^T f \\ &= -\frac{1}{2} f^T (C + X D^{-1} X^T) f + f^T X D^{-1} y - \frac{1}{2} y^T D^{-1} y. \end{aligned}$$

We next take the gradient with respect to f

$$\nabla_f \log p(f|y) = -(C + X D^{-1} X^T) f + X D^{-1} y,$$

and solve for $\hat{f}$ by setting $\nabla_f \log p(f|y) = 0$, which yields

$$\hat{f} = (C + X D^{-1} X^T)^{-1} X D^{-1} y. \tag{22}$$

If C is invertible, then we can define a kernel matrix $K = C^{-1}$ over the complete genotype-phenotype map and verify that this is equivalent to the classical solution for the posterior mean of a Gaussian process model using Woodbury's identity. Specifically, for a subset of sequences z and the α^ℓ by $|z|$ matrix Z defined by:

$$Z_{ij} = \begin{cases} 1 & \text{if sequence } i \text{ is the } j\text{-th member of } z \\ 0 & \text{otherwise,} \end{cases}$$

we find that

bioRxiv preprint doi: https://doi.org/10.1101/2025.03.09.642267; this version posted March 15, 2025. The copyright holder for this preprint (which was not certified by peer review) is the author/funder, who has granted bioRxiv a license to display the preprint in perpetuity. It is made available under aCC-BY 4.0 International license.

$$\begin{aligned}\hat{f}_z &= Z^T \hat{f} \\ &= Z^T (C + XD^{-1}X^T)^{-1} XD^{-1}y \\ &= Z^T \left(K - KX(X^T KX + D)^{-1} X^T K\right) XD^{-1}y \\ &= Z^T K \left(I - X(X^T KX + D)^{-1} X^T K\right) XD^{-1}y \\ &= Z^T K \left(X - X(X^T KX + D)^{-1} X^T KX\right) D^{-1}y \\ &= Z^T KX \left(I - (X^T KX + D)^{-1} X^T KX\right) D^{-1}y.\end{aligned}$$

Then we can use the fact that $I = (X^T KX + D)^{-1}(X^T KX + D)$ to obtain the identity:

$$\begin{aligned}&I - (X^T KX + D)^{-1} X^T KX \\ &= (X^T KX + D)^{-1}(X^T KX + D) - (X^T KX + D)^{-1} X^T KX \\ &= (X^T KX + D)^{-1}(X^T KX + D - X^T KX) \\ &= (X^T KX + D)^{-1} D\end{aligned}$$

Substituting this identity into our previous expression for $\hat{f}_z$, we now recover the classical maximum a posteriori solution for Gaussian process regression

$$\hat{f}_z = Z^T KX \left(X^T KX + D\right)^{-1} DD^{-1}y = Z^T KX \left(X^T KX + D\right)^{-1} y,$$

as desired.

Turning to the covariance matrix for the posterior, knowing that the posterior distribution is multivariate Gaussian implies that the posterior covariance matrix is given by the inverse of the Hessian matrix of the log-posterior probability. Thus the covariance matrix of the posterior distribution is given by:

$$\Sigma = \left(\nabla\nabla_f log(f|y)\right)^{-1} = \left(C + XD^{-1}X^T\right)^{-1}, \tag{23}$$

which we note depends on our observations only through the pattern of observed sequence as encoded in X and not on the observed phenotypes y.

Based on the marginalization property of multivariate Gaussian distributions, the posterior covariance at a subset of points z can be obtained simply by taking the submatrix $\Sigma_{zz} = Z^T \Sigma Z$. We can verify that this also matches the classical solution for Gaussian process posterior covariance when $K = C^{-1}$ using Woodbury's identity:

$$\begin{aligned}\Sigma_{zz} &= Z^T \left(C + XD^{-1}X^T\right)^{-1} Z \\ &= Z^T \left(C^{-1} - C^{-1}\left(X^T C^{-1} X + D\right)^{-1} C^{-1}\right) Z \\ &= Z^T \left(K - K\left(X^T KX + D\right)^{-1} K\right) Z \\ &= Z^T KZ - Z^T K \left(X^T KX + D\right)^{-1} KZ \\ &= K_{zz} - K_{zx}\left(K_{xx} + D\right)^{-1} K_{xz},\end{aligned} \tag{24}$$

as desired.
